# Engineering high-performance CTAB-functionalized magnesium silicate nano-adsorbent for efficient removal of Cd^2+^, Co^2+^, and Cu^2+^ from single-metal aqueous solutions

**DOI:** 10.3389/fchem.2025.1583305

**Published:** 2025-05-08

**Authors:** Marwa H. Shemy, Reham A. Mohamed, Ahmed A. Abdel-Khalek, Haifa A. Alqhtani, Wail Al Zoubi, Mostafa R. Abukhadra

**Affiliations:** ^1^ Department of Chemistry, Faculty of Science, Beni-Suef University, Beni-Suef City, Egypt; ^2^ Department of Biology, College of Science, Princess Nourah Bint Abdulrahman University, Riyadh, Saudi Arabia; ^**3**^ Materials Electrochemistry Laboratory, School of Materials Science and Engineering, Yeungnam University, Gyeongsan, Republic of Korea; ^4^ Materials Technologies and their Applications Lab, Geology Department, Faculty of Science, Beni-Suef University, Beni-Suef City, Egypt; ^5^ Applied Science Research Center, Applied Science Private University, Amman, Jordan

**Keywords:** serpentinite, organo-functionalization, CTAB, adsorption, heavy metals, advanced isotherm

## Abstract

The development of highly efficient, recyclable adsorbents for heavy metal remediation remains a critical challenge in environmental engineering. This study introduces a novel cetyltrimethylammonium bromide-functionalized magnesium silicate (CTAB/MS) nano-adsorbent was synthesized through a multi-step surface modification of serpentinite involving intercalation with dimethyl sulfoxide, methanol treatment, and CTAB incorporation. The resulting nanostructure was extensively characterized and applied for the removal of cadmium (Cd^2+^), cobalt (Co^2+^), and copper (Cu^2+^) ions from contaminated water. The characterization findings confirmed significant morphological and structural modifications, including enhanced surface area, functional group availability, and mesoporosity, which contributed to enhanced adsorption performance. The kinetic modeling confirmed that the process predominantly followed a pseudo-first-order model, suggesting that rapid physisorption mechanisms controlled the initial adsorption phase. Equilibrium studies revealed that adsorption followed the Langmuir isotherm model, indicating monolayer adsorption on homogeneous active sites, with maximum adsorption capacities of 491.9 mg/g (Cd^2+^), 481.8 mg/g (Co^2+^), and 434.3 mg/g (Cu^2+^) at 303 K. Furthermore, statistical physics-based isotherm model incorporating steric and energetic parameters provided deeper mechanistic insights. The adsorption energy (ΔE) values remained below 12.66 kJ/mol, confirming a predominantly physical adsorption process, while thermodynamic analysis indicated an exothermic and spontaneous nature, as evidenced by negative free enthalpy (G) and internal energy (E_int_) values. The recyclability assessment demonstrated that CTAB/MS retained over 70% of its adsorption efficiency after five consecutive regeneration cycles, underscoring its long-term applicability in water treatment. This highlights the potential of CTAB/MS as an advanced, cost-effective, and sustainable solution for large-scale water purification.

## 1 Introduction

The contamination of freshwater resources by chemical pollutants presents a critical challenge to environmental sustainability and public health, raising serious concerns about long-term ecological security (Yang et al., 2022). The uncontrolled discharge of highly contaminated effluents from industrial, agricultural, and mining activities remains a major contributor to water pollution and its associated environmental risks (Zheng et al., 2024a; Sharma et al., 2024). The presence of toxic metals in environments, whether in the form of dissolved ions or chemical complexes, poses significant threats to both ecological balance and human wellbeing (Zheng et al., 2024a; Sayed et al., 2022). These pollutants are characterized by their high toxicity, non-biodegradable nature, carcinogenic potential, and tendency to bioaccumulate in the tissues of living organisms, thereby exacerbating their long-term impact (Sayed et al., 2022; Alam et al., 2024; Jahin et al., 2022). Industries such as mining, manufacturing, and nuclear energy production are among the primary sources of hazardous metallic ions—including cadmium, zinc, chromium, mercury, iron, lead, manganese, and barium—discharged into natural water bodies (Kang et al., 2020; Park et al., 2023; Salam et al., 2020).

Cadmium (Cd^2+^) is particularly hazardous due to its extreme toxicity, and its concentration in drinking water should not exceed 0.003 mg/L, as stipulated by international regulatory standards (Vilela et al., 2019). Exposure to Cd^2+^ has been linked to a wide range of severe health effects, including pulmonary edema, acute and chronic illnesses, itai-itai disease, emphysema, liver failure, hypertension, testicular damage, kidney dysfunction, and osteomalacia (Souza-Arroyo et al., 2022; Rahman and Raheem, 2024). In addition to its harmful effects on human health, Cd^2+^ contamination poses a serious risk to agricultural productivity by significantly inhibiting seed germination, restricting crop growth, and impairing root elongation and leaf formation (El Rasafi et al., 2022). Furthermore, elevated cadmium concentrations in aquatic environments adversely affect marine biodiversity, threaten fish populations, and reduce the economic viability of fisheries (Sharma et al., 2024). Given these substantial risks, both the World Health Organization (WHO) and the American Water Works Association (AWWA) have set stringent guidelines, limiting Cd^2+^ concentrations in drinking water to a maximum of 0.005 mg/L to safeguard public health and environmental integrity (Chwastowski and Staroń, 2023).

Cobalt (Co^2+^) contamination in water sources primarily results from industrial activities such as mining, electroplating, and battery production, posing significant environmental and health hazards (De Oca-Palma et al., 2020; Díez et al., 2024). In aquatic ecosystems, excessive Co^2+^ levels disrupt enzymatic functions, induce oxidative stress in marine life, and accumulate in the food chain, threatening biodiversity (He et al., 2011). Chronic human exposure through contaminated water has been linked to cardiovascular diseases, thyroid dysfunction, respiratory disorders, and neurotoxicity (Rahman et al., 2023; Leyssens et al., 2017). Prolonged ingestion may also contribute to diabetes, reproductive toxicity, and developmental abnormalities (Rahman et al., 2023; Díaz-De-Alba et al., 2021). To mitigate these risks, the World Health Organization (WHO) recommends a maximum allowable concentration of cobalt 0.05 mg/L in drinking water (Dehghani et al., 2019). Such hazardous impacts were detected also as side effects for the over concentrations of coper (II) ions in the water supplies (Liu et al., 2023). Although copper is an essential micronutrient for humans, excessive levels can lead to severe toxicity (Varma and Misra, 2018). The World Health Organization (WHO) has set the permissible limit for copper in drinking water at 2 mg/L to prevent adverse health effects. The United States Environmental Protection Agency (USEPA) has fixed 1.3 mg/L as the tolerable limit of copper ions in drinking water (Manne et al., 2022; Wang F. et al., 2021). Exposure to elevated copper (II) concentrations can cause acute and chronic health issues. Short-term ingestion may result in gastrointestinal distress, nausea, vomiting, and diarrhea, while long-term exposure has been linked to liver and kidney damage, and neurotoxicity (Wang F. et al., 2021; Wang et al., 2022). In aquatic environments, excessive copper can disrupt ecosystems by impairing the metabolic functions of fish, invertebrates, and microorganisms, leading to bioaccumulation and biomagnification in the food chain (Wang X. et al., 2021).

Numerous studies have identified adsorption using advanced materials as a highly efficient, cost-effective, sustainable, and recyclable strategy for eliminating various contaminants from water sources (Ashraf et al., 2022; Albukhari et al., 2021). The selection of an optimal adsorbent is influenced by multiple factors, including production costs, synthesis methods, precursor availability, adsorption capacity, reusability, adsorption kinetics, biodegradability, selectivity, safety, durability, and chemical reactivity (Sharma et al., 2024; Zheng et al., 2024b). As a result, extensive research has been dedicated to developing innovative adsorbents utilizing naturally abundant and low-cost materials (Yang et al., 2022; Ghonim et al., 2022; Sun et al., 2024). The use of adsorbents derived from natural resources, such as minerals and rocks, is strongly advocated due to their economic feasibility and environmental sustainability (Chen et al., 2021a). Extensive research efforts have been undertaken to design innovative adsorbents utilizing readily available and cost-effective materials commonly found in natural resources (Ifa et al., 2022; Hamidi et al., 2023). The development and application of adsorbents derived from earth’s resources, including various minerals and rocks, are strongly recommended due to their substantial environmental and economic advantages (Chen et al., 2021b).

Clay-based nanomaterials have been widely recognized for their effectiveness in safely removing a broad spectrum of organic and inorganic contaminants, offering the dual benefits of affordability and environmental sustainability (Alqahtani et al., 2023; Yao et al., 2024; Ahmed et al., 2024). These clay minerals typically consist of layered aluminosilicate structures with flexible frameworks, making them highly adaptable for adsorption applications. They are characterized by strong ion-exchange capabilities, chemical stability, high thermal resistance, and a reactive surface that facilitates chemical interactions (Alqahtani et al., 2023; Ighnih et al., 2023a; Wang et al., 2013). To enhance the surface chemistry and physicochemical properties of widely used clay minerals, various modification techniques have been explored to optimize their performance as adsorbents for removing organic and inorganic pollutants (Ighnih et al., 2023a; Meng et al., 2020). These modification approaches include alkaline chemical treatments, thermal processing, acidic activation, metal-ion pillaring, incorporation of metal oxides, exfoliation, polymer integration, scrolling, and organic hybridization using reagents such as cetyltrimethylammonium bromide (CTAB) and starch (Ighnih et al., 2023a; Meng et al., 2020; Ighnih et al., 2023b).

Despite extensive research on clay mineral exfoliation and organo functionalization, most studies have predominantly focused on aluminosilicate-based clays such as bentonite, glauconite, and kaolinite (Zhu et al., 2022; Das et al., 2023). Limited attention has been given to magnesium-rich clay minerals like talc and serpentinite, which differ structurally due to their magnesium-dominated composition rather than aluminum (Wang et al., 2023; Chen et al., 2024). Serpentinite (Mg_3_Si_2_O_5_(OH)_4_) belongs to the phyllosilicate mineral group, featuring a 1:1 layered structure with alternating tetrahedral Si–O and octahedral Mg–OH layers (Wang et al., 2023; Chen et al., 2024). Its structure contains abundant hydroxyl (-OH) functional groups and surface-bound water molecules, contributing to its moderate adsorption capacity (Martínez et al., 2024; Basit et al., 2024). However, its inherently limited pore volume, surface area, and interlayer spacing restrict its broader application in water treatment (Chen et al., 2024; Chen M. et al., 2021; Bharali, 2024).

To overcome these limitations, this study presents an advanced modification approach by intercalating serpentinite with cetyltrimethylammonium bromide (CTAB), forming partially exfoliated organo-functionalized magnesium silicate nanosheets (CTAB/MS) with enhanced adsorption efficiency for heavy metal ions. The adsorption performance of CTAB/MS was systematically evaluated for Cd^2+^, Co^2+^, and Cu^2+^ removal under various experimental conditions. Additionally, adsorption kinetics, classical isotherm models, and advanced statistical physics-based isotherm modeling were applied to gain deeper insights into adsorption mechanisms. The steric parameters examined included saturation capacity, reactive site density, and the occupancy rate per active site. Meanwhile, the energetic investigation addressed adsorption energy and key thermodynamic functions, providing a comprehensive understanding of the adsorption process.

## 2 Experimental work

### 2.1 Materials

The Serpentinite mineral (Mg_3_Si_2_O_5_(OH)_4_) utilized in this study was obtained from serpentine quarry, Eastern desert, Egypt. To aid the modifications process, chemicals procured from Sigma-Aldrich, Egypt, were employed, including dimethyl sulfoxide (DMSO; 99.5%) serving as both a solvent and swelling agent, methanol (99.9%), and cetyltrimethylammonium bromide (CTAB; 98%). During adsorption studies, cadmium nitrate tetrahydrate (Cd(NO_3_)_2_ 4H_2_O; 99%), cobalt nitrate hexahydrate (Co(NO_3_)_2_ 6H_2_O; 97%), copper nitrate trihydrate (Cu(NO_3_)_2_ 3H_2_O; 95%), sodium hydroxide (NaOH; 97%), and nitric acid (HNO_3_; 55%) were used.

### 2.2 Synthesis of serpentinite based oregano functionalized magnesium silicate (CTAB/MS)

**FIGURE 1:**
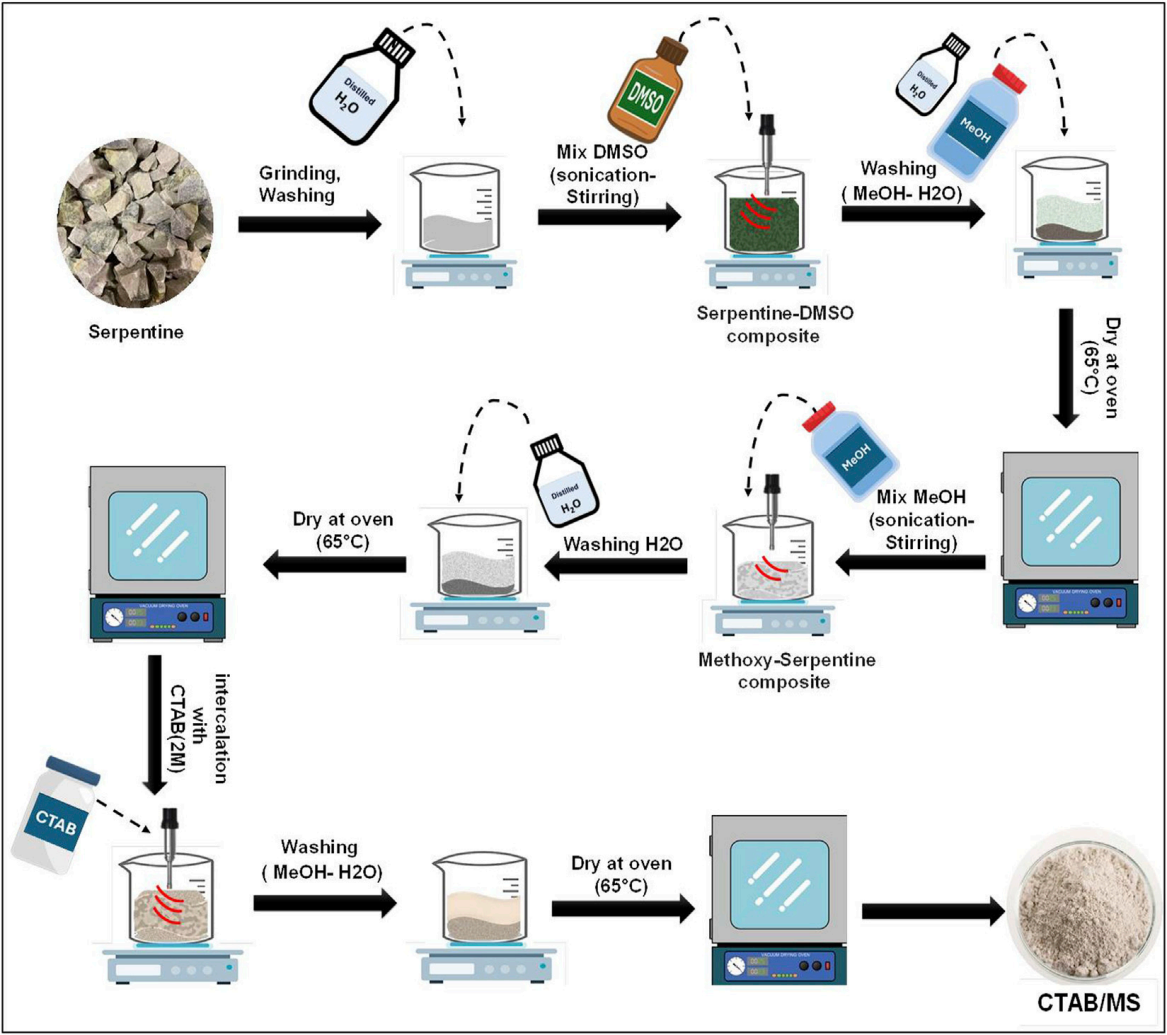
Schematic diagram for the synthesis procedures of CTAB/MS nanostructure.

The modification process was performed using a three-step process considering the structural properties of serpentinite as magnesium silicate sheets connected by strong hydrogen bond. Initially, the raw mineral was mechanically ground and subjected to stirring in distilled water at 600 rpm for 60 min. The next phase involved mixing the sample with DMSO at a ratio of 1 g (solid)/1 mL (DMSO), followed by alternating stirring and sonication for 72 h to facilitate DMSO penetration into the clay layers, thereby promoting the destruction of hydrogen bonds. The resultant serpentinite-DMSO complex (Ser-DMSO) underwent multiple washes using a 1:1 methanol-distilled water solution, each lasting 20 min at 600 rpm, and was subsequently dried at 65°C. In the second stage, the Ser-DMSO complex was immersed in methanol (1 g: 10 mL) for 72 h, alternating between stirring at 50°C (1,500 rpm) and sonication (240 W). Following five successive washes with distilled water (each for 20 min at 600 rpm), the material was dried at 65°C. The final stage involved immersing the methoxy-treated serpentine in a 2 M CTAB solution for 72 h, with vigorous stirring at 50°C (1,500 rpm), alternated with sonication (240 W) and vibration (50 Hz) for 8 h daily. The resultant material was washed repeatedly with a 1:1 distilled water-methanol solution, filtered, and dried at 65°C, yielding highly hybrid stripped and partially exfoliated organo functionalized magnesium silicate structure (CTAB/MS) (Figure 1).

### 2.3 Characterization techniques

The surface morphology and structural attributes of the synthesized materials, along with the precursor Serpentine, were examined using a Gemini Zeiss Ultra 55 scanning electron microscope (SEM). Prior to imaging, the samples were coated with gold films to enhance resolution. Additionally, the internal morphologies were investigated using high-resolution transmission electron microscopy (HRTEM) *via* a JEOL-JEM2100 microscope operating at 200 kV. The specific surface area and porosity were analyzed using a Beckman Coulter SA3100 surface area analyzer, following specimen degassing. Surface area estimations were performed using the Brunauer–Emmett–Teller (BET) method, while pore characteristics were determined using the Barrett–Joyner–Halenda (BJH) method through nitrogen adsorption-desorption isotherms. Structural and chemical transformations across synthesis phases were examined *via* Fourier Transform Infrared Spectroscopy (FTIR) using a Shimadzu FTIR-8400S spectrometer, covering a detection range of 400–4,000 cm^−1^. X-ray diffraction (XRD) analysis was employed to assess crystallinity and structural features of the CTAB/MS, utilizing a PANalytical-Empyrean X-ray diffractometer with a scanning range from 0° to 70°. Crystallite size was estimated using the Scherrer equation, while d-spacing of the (001) plane was determined using Bragg’s Law.

### 2.4 Batch adsorption studies of Cd^2+^, Co^2+^, and Cu^2+^ metal ions

Batch adsorption experiments were conducted to evaluate the uptake of Cd^2+^, Co^2+^, and Cu^2+^ ions using CTAB/MS. The study considered various parameters, including solution pH (2–8), contact time (0.5–36 h), and initial metal ion concentrations (50–350 mg/L). Temperature was systematically adjusted to 303 K, 313 K, and 323 K, while volume (100 mL), CTAB/MS dosage (0.02 g/L), pH (6), and temperature (303 K) were maintained constant. Each experiment was performed in triplicate, and mean concentration values were analyzed. Post-equilibration, metal-laden CTAB/MS were separated using Whatman filter papers (40 μm), and residual ion concentrations were measured using inductively coupled plasma mass spectrometry (ICP-MS). Adsorption capacity (Qe) and removal efficiency were determined using Equations (1) and (2):

$$Q_{e\;\left(mg/g\right)}=\frac{\left(C_{o}-C_{e}\right)V}{m}$$ (1)

$$R\text{emoval},\left(\%\right)=\frac{100\;X\;\left(C_{o}-C_{e}\right)}{C_{o}}$$ (2)

### 2.5 Quality assurance and quality control

To ensure the accuracy and reliability of experimental results, all analytical procedures were carried out following standard quality assurance and quality control (QA/QC) protocols. Each adsorption experiment was performed in triplicate, and the reported results represent the average values with standard deviations maintained below 5%, confirming good reproducibility. Analytical instruments, including inductively coupled plasma mass spectrometry (ICP-MS), were calibrated using certified multi-element standard solutions prior to each measurement session. Calibration curves were constructed with correlation coefficients (*R* ^2^) exceeding 0.998. Procedural blanks and control samples were routinely analyzed alongside test samples to check for background contamination or instrumental drift. The pH meter was regularly calibrated with standard buffer solutions (pH 4, 7, and 10), and temperature control was maintained using a thermostatic shaker bath with ±0.5 °C accuracy. Reagents and solvents used were of analytical grade and handled under contamination-free conditions. All glassware was thoroughly acid-washed and rinsed with deionized water before use. These practices collectively ensured the precision, accuracy, and reproducibility of the data generated in this study.

### 2.6 Equilibrium investigations: Traditional and advanced approaches

Adsorption behavior was modeled using various approaches, including classical kinetics and isotherm analyses, along with advanced equilibrium models based on statistical physics. The models included Pseudo-First-Order and Pseudo-Second-Order kinetics, as well as Langmuir (L.G), Freundlich (F.E), and Dubinin-Radushkevich (D-R) isotherms (Supplementary Table S1). Model fitting was achieved through nonlinear regression, evaluated using correlation coefficients (*R* ^2^) and Chi-squared (χ^2^), as expressed in Equations (3) and (4):

$$R^{2}=1-\frac{\sum {\left(q_{e,\exp}-q_{e,cal}\right)}^{2}}{\sum {\left(q_{e,\exp}-q_{e,mean}\right)}^{2}}$$ (3)

$$\chi^{2}=\sum \frac{{\left(q_{e,\exp}-q_{e,cal}\right)}^{2}}{q_{e,cal}}$$ (4)

Further analysis incorporated the coefficient of determination (*R* ^2^) and root-mean-square error (RMSE), calculated *via* Equation (5):

$$RMSE=\sqrt{\frac{\sum_{i=1}^{m}{\left({Qi}_{cal}-{Qi}_{\exp}\right)}^{2}}{m^{\prime }-p}}$$ (5)

Adsorption mechanisms were elucidated through steric and energetic parameters such as adsorption energy (Δ*E*), entropy (Sa), internal energy (*E*_int_), and enthalpy (*G*), derived using Equations 6–9. These equations provided insights into the adsorption thermodynamics of Cd^2+^, Co^2+^, and Cu^2+^ ions onto CTAB/MS.

$$\Delta E=\text{RT In}\frac{s}{c}$$ (6)

Entropy values (*S*_*a*_) are deduced from Equation (6), wherein the supplied entropy (Sa) is proportional to the product of active site density (Nm) and a function involving the concentration of organic compounds (C), the half-saturation concentration (C_1/2_), and the number of molecules per site (n). Additionally, utilizing the steric parameters and the translation partition value (Zv), Equations (8) and (9) are utilized to determine the internal energy (*E*_int_) and enthalpy (*G*), respectively. These equations unveil the intricate connections among steric variables, concentration levels, and thermodynamic parameters, offering essential comprehension into the adsorption mechanisms governing Cu^2+,^ Cd^2+^, and Co^2+^ ions in CTAB/MS systems.

$$\frac{S_{a}}{K_{B}}=Nm\left\{\ln\left(1+{\left(\frac{C}{C_{\frac{1}{2}}}\right)}^{n}\right)-n{\left(\frac{C}{C_{\frac{1}{2}}}\right)}^{n}\;\frac{\ln\left(\frac{C}{C_{\frac{1}{2}}}\right)}{1+{\left(\frac{C}{C_{\frac{1}{2}}}\right)}^{n\;}}\;\right\}$$ (7)

$$\frac{E_{\mathrm{int}}}{K_{B}T\;}=n\;N_{m}\left[\left(\;\frac{{\left(\frac{C}{C_{\frac{1}{2}}}\right)}^{n}\;\ln\left(\frac{C}{Z_{v}}\right)}{1+{\left(\frac{C}{C_{\frac{1}{2}}}\right)}^{n}}\right)-\left(\;\frac{n\ln\left(\frac{C}{C_{\frac{1}{2}}}\right)\;{\left(\frac{C}{C_{\frac{1}{2}}}\right)}^{n}}{1+{\left(\frac{C}{C_{\frac{1}{2}}}\right)}^{n}}\right)\right]$$ (8)

$$\frac{G}{K_{B}T\;}=nN_{m}\frac{\ln\left(\frac{C}{Z_{v}}\right)}{1+{\left(\frac{C_{\frac{1}{2}}}{C}\right)}^{n}}$$ (9)

## 3 Results and discussion

### 3.1 Characterization of CTAB/MS

#### 3.1.1 XRD analysis

**FIGURE 2:**
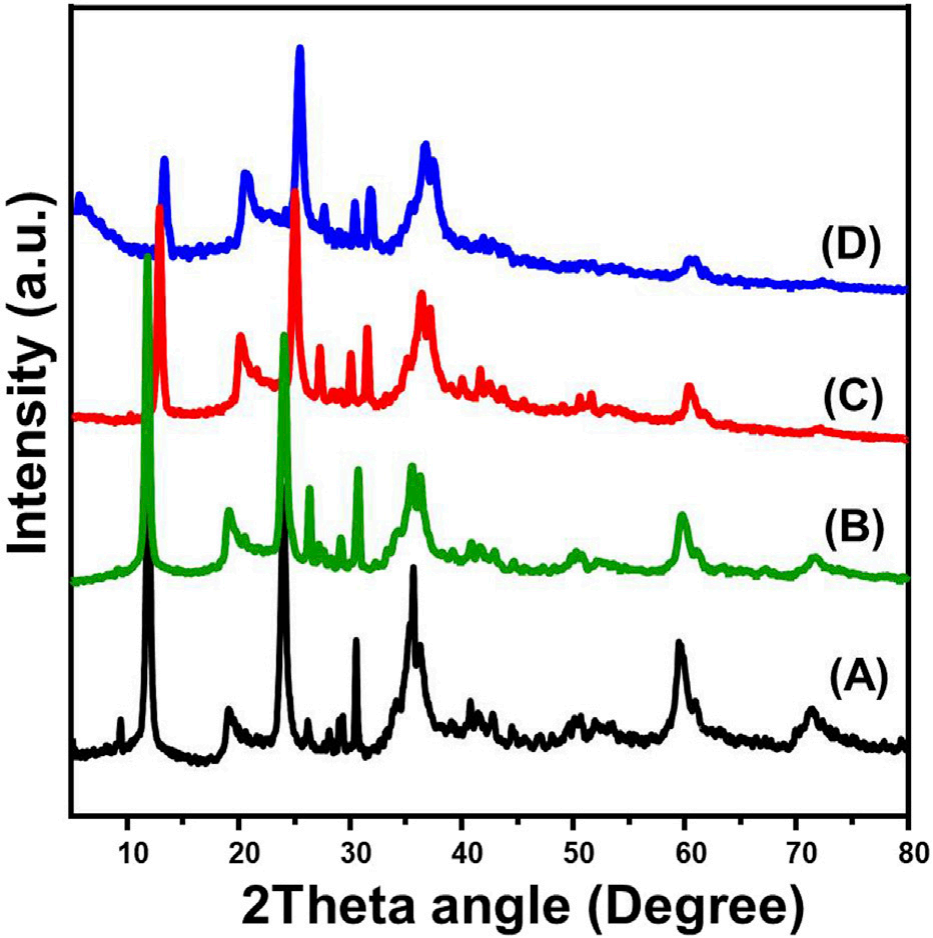
XRD patterns of raw serpentinite **(A)**, DMSO-serpentinite **(B)**, methoxy serpentinite **(C)**, and CTAB modified serpentinite **(D)**.

X-ray diffraction (XRD) analysis confirmed the presence of serpentine in two well-defined crystalline polymorphs, antigorite and chrysotile, within the examined sample (Figure 2). The predominance of antigorite was evident from the distinct diffraction peak corresponding to the (001) crystallographic plane at 2θ = 12°, accompanied by characteristic reflections at 24° (004) and 35.85° (−131), with a measured d-spacing of 0.72 nm, aligning with previously reported data (XRD Cd. No. 00-007-0417) (Figure 2A). In contrast, the presence of chrysotile was confirmed by the detection of diffraction peaks at 9.5°, 12.1°, 19.7°, 24.2°, and 60°, corresponding to the XRD reference pattern (Figure 2A) (XRD Cd. No. 00-010-0380). The structural integrity of serpentine was significantly altered following dimethyl sulfoxide (DMSO) intercalation, as reflected by pronounced peak shifts and broadening effects in the XRD pattern. The characteristic diffraction peaks of serpentine underwent noticeable displacements at 2.9°, 12.8°, 20.5°, 25.1°, 28°, 31.5°, 36.6°, and 60.6°, indicating substantial changes in the crystalline lattice due to guest molecule incorporation (Figure 2B) (Shawky et al., 2019; Abukhadra et al., 2024). Moreover, the disappearance of the original peak at 9.3°, which was present in the untreated serpentine, suggested a crystallinity disruption caused by DMSO penetration into the silicate layers. This structural alteration is characteristic of successful intercalation, leading to layer expansion and modification of the material’s physicochemical properties.

Subsequent methoxy functionalization further impacted the XRD profile of serpentine, as evident from peak shifts and intensity variations (Figure 2C) (Abukhadra et al., 2024; Ashiq et al., 2021). This transformation was attributed to the formation of hydrogen bonds, which are typically observed in methoxy-modified silicates following DMSO pretreatment (Figure 2C). The introduction of methoxy groups likely altered the interlayer interactions, influencing both basal spacing and crystallinity, thus enhancing the material’s potential for advanced applications in adsorption and catalysis (Ashiq et al., 2021; Tan et al., 2017). The final phase of modification involved the intercalation of cetyltrimethylammonium bromide (CTAB), which induced further structural reorganization within the serpentine framework (Figure 2D). A key observation was the significant shift of the (001) peak to approximately 11.7°, providing strong evidence of CTAB molecule penetration into the magnesium silicate layers (Abukhadra et al., 2024; Guo et al., 2020). This intercalation process is particularly relevant for surface functionalization and enhanced dispersion, which are critical factors for adsorption efficiency, and surface reactivity.

The XRD investigations provide comprehensive insights into the structural evolution of serpentine subjected to various chemical modifications. Initially, the pristine sample was dominated by antigorite, with chrysotile appearing in minor quantities. The introduction of DMSO followed by methanol and CTAB molecules led to layer expansion, peak broadening, and crystallinity reduction, confirming successful intercalation and lattice modification. These findings underscore the versatility of serpentine as a modifiable magnesium silicate platform. The observed transformations through intercalation and chemical treatment highlight its potential for nanomaterials engineering, catalytic applications, and adsorption-based technologies. By controlling interlayer spacing and crystallinity, chemically modified serpentine can be tailored for enhanced reactivity, surface area optimization, and selective adsorption properties, making it a promising material for environmental remediation.

#### 3.1.2 FT-IR analysis

**FIGURE 3:**
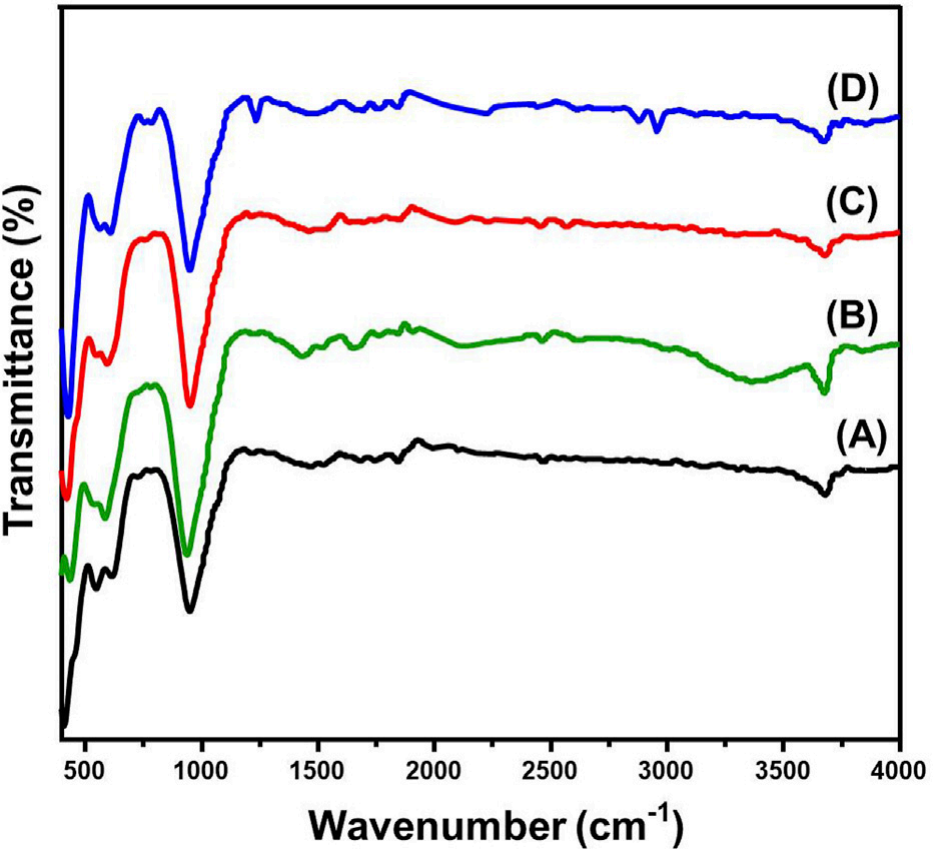
FT-IR spectra of raw serpentinite **(A)**, DMSO-serpentinite **(B)**, methoxy serpentinite **(C)**, and CTAB modified serpentinite **(D)**.

Fourier Transform Infrared (FT-IR) spectroscopy was employed to investigate the structural characteristics, functional group interactions, and chemical modifications of serpentinite. The obtained FT-IR spectra provided critical insights into the mineral’s intrinsic vibrational features and its structural transformations following intercalation and functionalization (Figure 3). The FTIR spectrum of untreated serpentinite exhibited characteristic vibrational bands corresponding to hydroxyl stretching, Si–O–Si lattice vibrations, and Mg–OH bending, confirming its layered silicate framework (Figure 3A) (Altowyan et al., 2022). A prominent absorption peak at 3,671 cm^−1^ was assigned to structural hydroxyl (-OH) groups, which are either coordinated to magnesium (Mg–OH) within the octahedral layers or involved in hydrogen bonding within the silicate lattice (Crespo et al., 2019; Leask et al., 2021). The silicate framework vibrations were represented by bands in the 900–1,000 cm^−1^ region, attributed to Si–O–Si and Si–O stretching modes, characteristic of phyllosilicates (Figure 3A) (Crespo et al., 2019). Additionally, absorption bands in the 400–900 cm^−1^ range were assigned to Mg–OH bending vibrations, indicative of interactions between hydroxyl groups and magnesium in the octahedral layers, alongside Mg–O stretching modes, confirming the serpentine mineralogy (Leask et al., 2021).

Intercalation of dimethyl sulfoxide (DMSO) into the serpentinite structure resulted in significant spectral shifts, indicating strong molecular interactions and alterations in crystallinity (Figure 3B). The hydroxyl stretching band at 3,600 cm^−1^ exhibited a downshift, suggesting the formation of hydrogen bonds between the sulfonyl (-S=O) groups of DMSO and the inner metal-OH groups of the silicate layers (Figure 3B) (Abukhadra et al., 2024; Wang J. et al., 2021). Furthermore, a new peak at 3,300 cm^−1^ emerged, attributed to C-H stretching vibrations from DMSO or hydrogen bonding between the sulfonyl oxygen of DMSO and surface hydroxyl groups (Figure 3B) (de Macêdo Neto et al., 2015). The presence of a distinct absorption band at 1,427 cm^−1^ confirmed the vibrational signature of methyl (-CH_3_) groups, further validating the successful intercalation process (Wang J. et al., 2021). The silicate framework also exhibited notable changes, with the Si–O–Si and Si–O stretching vibrations undergoing broadening and slight peak shifts, indicative of increased structural disorder due to DMSO intercalation.

Subsequent methoxy functionalization led to additional modifications in the FTIR spectrum, suggesting substantial changes in the surface chemistry and structural organization of serpentinite (Figure 3C). A reduction in the intensity of hydroxyl stretching bands indicated the partial replacement of surface OH groups with methoxy (-OCH_3_) functionalities, forming Si–O–C bonds with methanol (Matusik et al., 2012; Khan and Wiley, 2022). Additionally, shifts in the Si–O–Si and Si–O stretching bands provided evidence of silicate layer reorganization due to methanol interaction. The disappearance of bands at 1,427 cm^−1^ and 3,300 cm^−1^ confirmed the complete displacement of intercalated DMSO molecules by methanol, further validating the chemical transformation process (Khan and Wiley, 2022; Qu et al., 2019).

Intercalation with cetyltrimethylammonium bromide (CTAB) resulted in additional spectral modifications, reflecting the incorporation of surfactant molecules within the silicate matrix (Figure 3D). The appearance of two intense peaks at 2,860 cm^−1^ and 2,930 cm^−1^ corresponded to symmetric and asymmetric C-H stretching vibrations of CTAB’s alkyl chains, confirming its successful incorporation into the serpentine structure (Figure 3D) (Abukhadra et al., 2024; Asadi et al., 2021). A shift in the Si–O stretching region (1,000–1,100 cm^−1^) indicated interlayer expansion due to surfactant intercalation. Moreover, the emergence of a distinct peak in the 1,470–1,490 cm^−1^ range, attributed to N–CH_3_ bending vibrations, further validated the interaction between CTAB and the serpentine matrix (Figure 3D) (Rumiyanti et al., 2025). The observed spectral shifts, intensity variations, and new vibrational features provide compelling evidence of successful intercalation and functionalization processes, leading to a tailored modification of serpentinite’s surface properties. The DMSO intercalation step introduced strong hydrogen bonding interactions, inducing structural disorder, while methoxy functionalization facilitated further molecular substitution and layer reorganization. Finally, CTAB intercalation significantly altered the surface chemistry, promoting the formation of an organic-modified serpentine phase. These structural transformations highlight serpentinite’s versatility for advanced applications in adsorption where controlled surface properties play a critical role in enhancing performance.

#### 3.1.3 EDX analysis

The elemental composition of serpentinite and its modified derivatives was assessed using energy-dispersive X-ray (EDX) spectroscopy, providing quantitative insight into the impact of chemical modifications on the material’s structure (Supplementary Figure S1). The pristine serpentinite exhibited a typical silicate composition, predominantly consisting of oxygen (O) (49%), magnesium (Mg) (26.7%), and silicon (Si) (21.3%), with minor contributions from iron (Fe) (1.2%), aluminum (Al) (1%), carbon (C) (0.7%), and sulfur (S) (0.1%) (Supplementary Figure S1). This elemental distribution is consistent with the expected magnesium silicate framework characteristic of serpentine minerals. Following the final modification step, where cetyltrimethylammonium bromide (CTAB) was intercalated into the silicate structure (CTAB/MS), noticeable alterations in the elemental composition were observed. The proportion of oxygen (O) decreased to 42.5%, likely due to the replacement of surface hydroxyl groups and the introduction of hydrophobic CTAB molecules. In contrast, carbon (C) content significantly increased to 22.6%, confirming the incorporation of the organic surfactant (Supplementary Figure S1). Other elemental proportions also changed: magnesium (Mg) was reduced to 15.7%, silicon (Si) decreased to 14.7%, while iron (Fe) and aluminum (Al) slightly increased to 2.9% and 1.6%, respectively. Notably, sulfur (S) was no longer detected, suggesting either its removal during the functionalization process or its insignificant presence after CTAB modification (Supplementary Figure S1). These findings provide substantial evidence of successful surface modification, with CTAB intercalation leading to organic incorporation and structural reorganization. The observed changes in elemental distribution further indicate a modification of the mineral’s surface chemistry, which can directly influence its adsorption behavior, hydrophobicity, and interfacial interactions in various applications.

#### 3.1.4 SEM analysis

**FIGURE 4:**
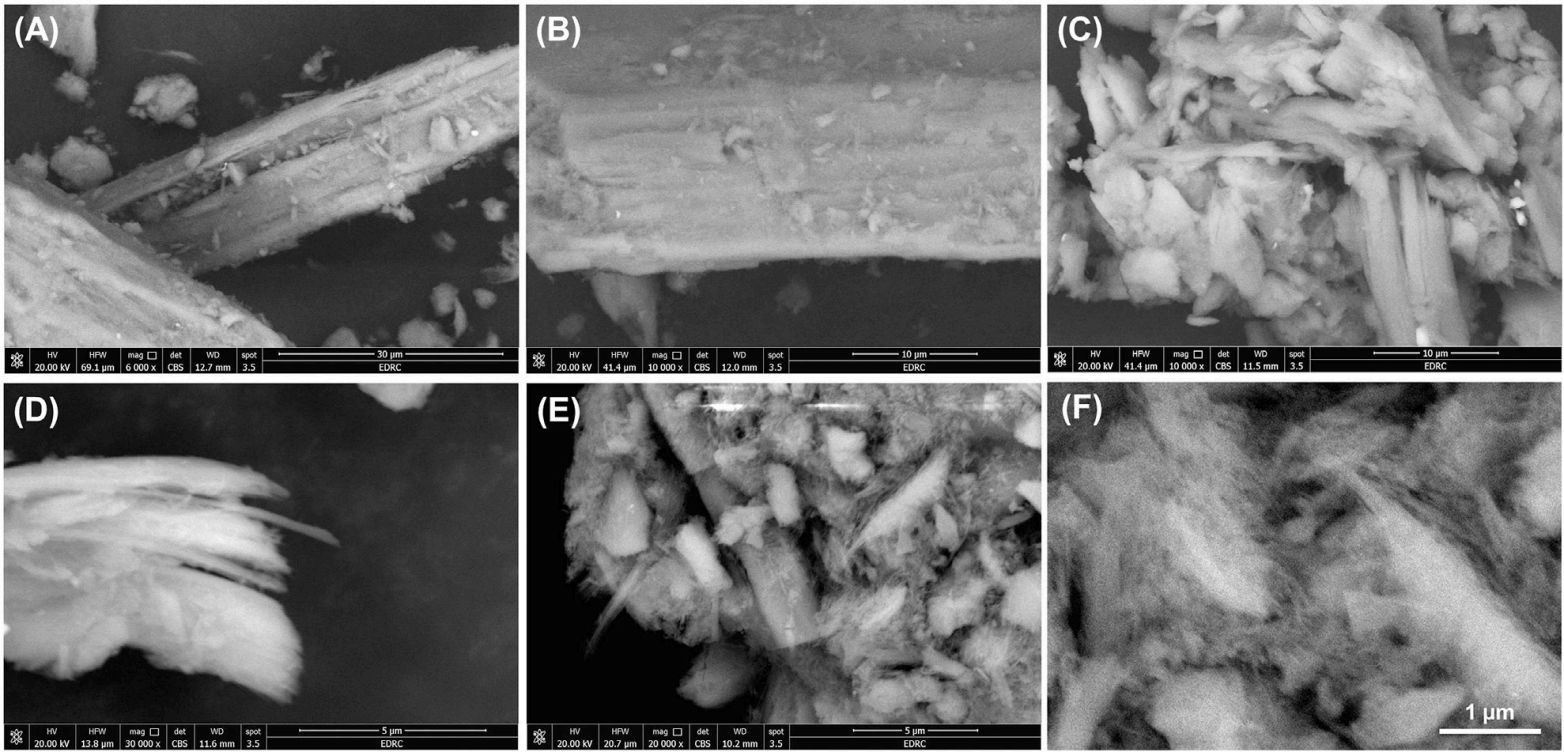
SEM images raw serpentinite **(A and B)**, DMSO-serpentinite **(C)**, methoxy serpentinite **(D)**, and CTAB modified serpentinite **(E and F)**.

The scanning electron microscopy (SEM) analysis provides critical insights into the morphological evolution of serpentinite during different stages of chemical modification. The unmodified serpentinite exhibits its intrinsic morphology, characterized by elongated, fibrous, and platy structures, which are typical of layered silicate minerals (Figure 4A). The surface of the serpentinite appears relatively smooth with minimal particle attachment, indicative of its well-ordered crystalline structure (Figures 4A,B). Upon undergoing initial chemical modifications, including dimethyl sulfoxide (DMSO) and methanol intercalation, noticeable alterations in morphology are observed. The SEM images reveal a progressive fragmentation and partial exfoliation, leading to enhanced exposure of the internal layers of the mineral (Figures 4C,D). The formation of more pronounced elongated platelets and sheet-like structures (Figure 4C) suggests that the intercalation process disrupts the layered architecture of serpentinite, promoting its structural disintegration. Following methanol intercalation, the material undergoes further exfoliation and loosening of the platy structures, resulting in thin, loosely packed and elongated platelets with increased surface roughness (Figure 4D). This confirms the strong impact of intercalation reactions on the mineral’s texture and morphology.

In the final modification step, cetyltrimethylammonium bromide (CTAB) intercalation induces significant structural transformations. The originally partially exfoliated platelets or fibrous structures undergo extensive delamination, forming nano-sized fibrils of CTAB-functionalized serpentinite or magnesium silicate (Figures 4E,F). These drastic morphological changes confirm the efficiency of surfactant intercalation in promoting exfoliation and increasing surface disorder. The observed morphological modifications directly influence the textural properties of the material, enhancing its surface area and reactivity. Such structural alterations are critical for improving the adsorption capacity and interfacial interactions of serpentinite in various applications.

#### 3.1.5 Textural properties

**FIGURE 5:**
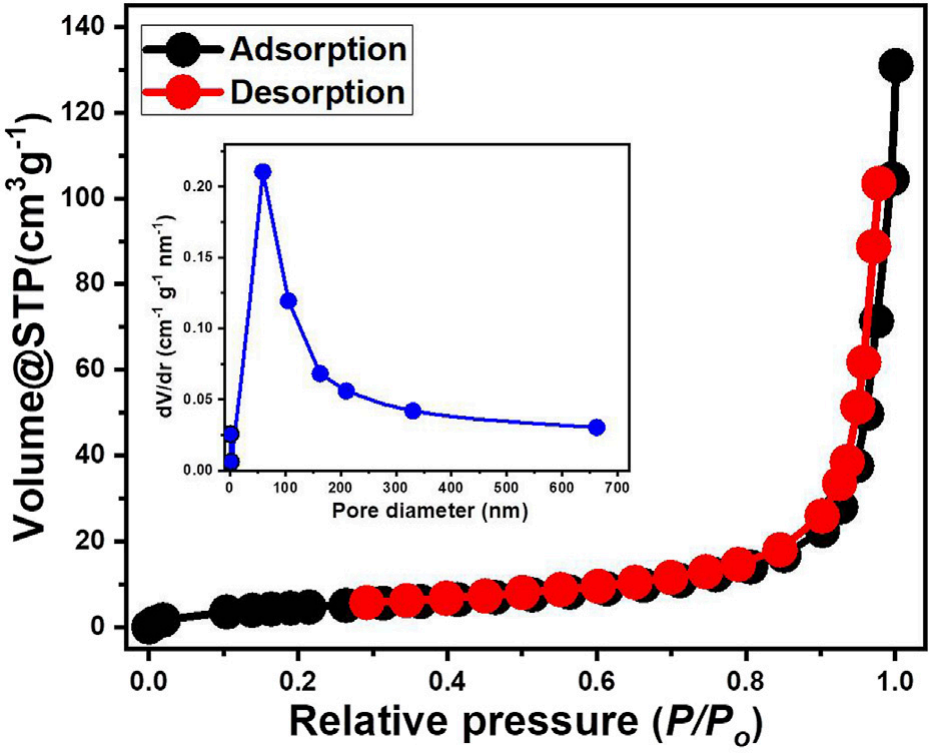
The nitrogen adsorption/desorption isotherm curve and pore size distribution of CTAB/MS structure.

The nitrogen adsorption-desorption isotherm curve (Figure 5) offers valuable insights into the porosity, surface characteristics, and textural properties of the synthetic CTAB/MS particles. The obtained isotherm exhibits a distinctive sigmoidal profile, where the adsorption and desorption branches overlap at lower relative pressures but begin to diverge significantly at higher pressures. Based on the International Union of Pure and Applied Chemistry (IUPAC) classification, this behavior is characteristic of a Type IV isotherm, which is commonly associated with mesoporous materials. A defining feature of this Type IV isotherm is the presence of an initial monolayer and multilayer adsorption process occurring at low relative pressures, followed by capillary condensation at higher pressures, which confirms the existence of well-defined mesopores. The hysteresis loop observed between adsorption and desorption further substantiates the mesoporous nature of the material, indicating pore connectivity and accessibility. This structural attribute enhances mass transport and diffusion properties, making the material a promising candidate for adsorption, catalysis, and separation processes.

Additionally, the pore size distribution curve (inset of Figure 5) provides further confirmation of the mesoporous framework, with a predominant pore size below 100 nm, followed by a gradual decrease as the diameter increases. The high specific surface area (17.38 m^2^/g) of the synthetic CTAB/MS particles suggests an extensive mesoporous network, which significantly enhances the adsorption capacity of the material. The observed isotherm behavior also reflects the structural stability of the modified material, confirming that the synthetic modifications did not compromise the pore architecture but instead improved its textural properties. Furthermore, the pronounced increase in adsorption at higher relative pressures highlights the potential applicability of CTAB/MS particles in a range of advanced functional applications, including drug delivery systems, heterogeneous catalysis, and gas storage technologies. The combination of high surface area, mesoporosity, and robust structural integrity ensures that the material possesses enhanced performance capabilities for various industrial and environmental applications.

### 3.2 Adsorption results

#### 3.2.1 Influence of pH

**FIGURE 6:**
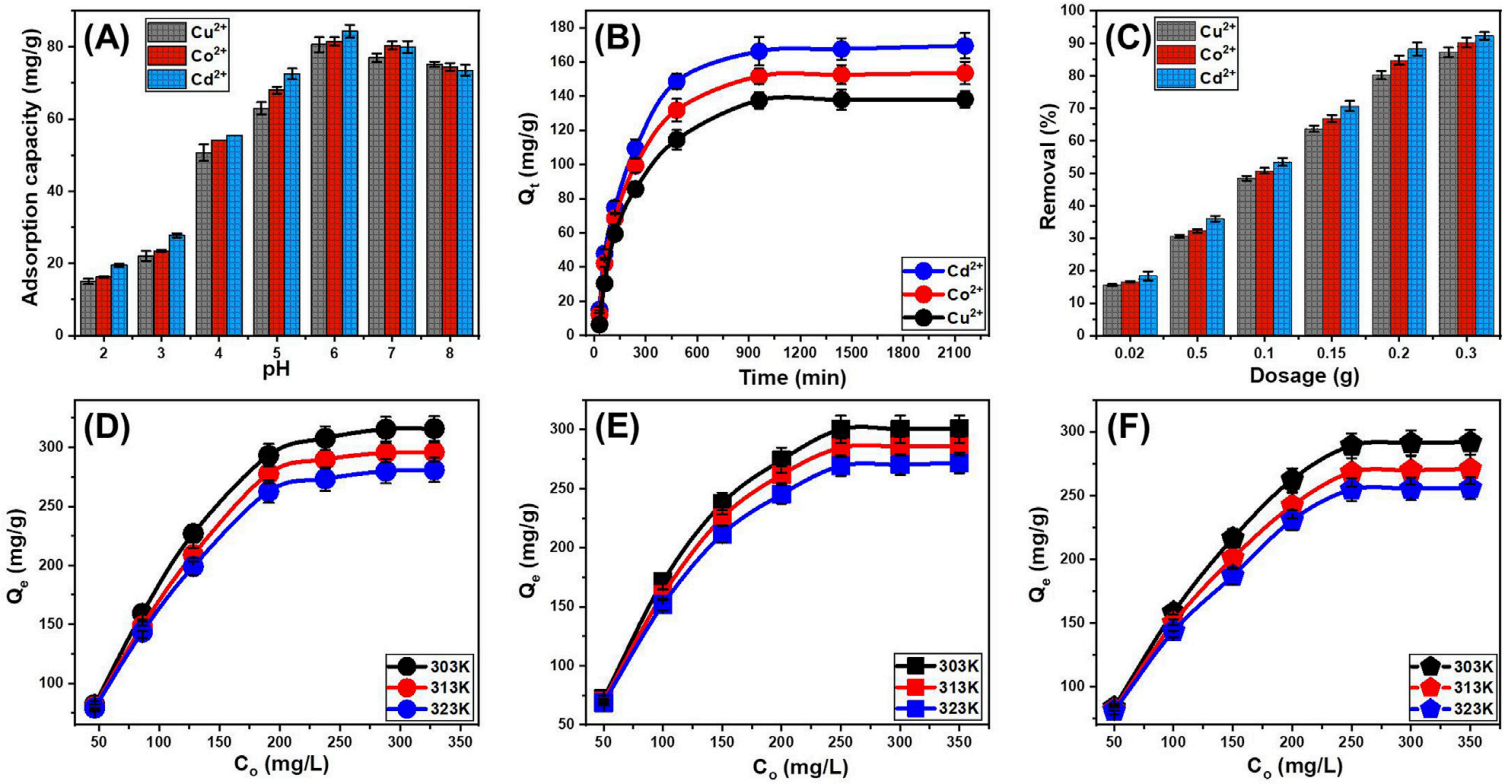
The experimental effect of the main factors on the adsorption of the metal ions by CTAB/MS structure including the pH values **(A)**, contact time **(B)**, CTAB/MS dosage **(C)**, and starting concentrations of the metals [Cd^2+^ **(D)**, Co^2+^ **(E)**, and Cu^2+^ **(F)**].

The pH of the solution plays a pivotal role in metal ion adsorption processes by influencing surface charge properties, metal speciation, competitive interactions, and adsorption site accessibility. Understanding the pH-dependent behavior of synthetic serpentine nanoparticles (CTAB/MS) in removing Cd^2+^, Co^2+^, and Cu^2+^ is essential for optimizing adsorption efficiency and ensuring practical applicability in wastewater treatment. In this study, adsorption experiments were conducted across a pH range of 2 to 8, with the upper limit constrained to prevent metal hydroxide precipitation (Figure 6). The experiments were performed under standardized conditions: 100 mg/L initial metal concentration, 0.02 g/L adsorbent dosage, 100 mL solution volume, 30°C temperature, and 120 min contact time.

At a highly acidic pH of 2, adsorption capacities were relatively low: 19.55 mg/g for Cd^2+^, 16.25 mg/g for Co^2+^, and 15 mg/g for Cu^2+^ (Figure 6A). This poor adsorption efficiency can be primarily attributed to the competition between metal cations and excess protons (H^+^) for active binding sites on the CTAB/MS surface. At such a low pH, the surface charge of the nanoparticles remains highly protonated, leading to increased electrostatic repulsion between the positively charged surface and the metal cations (Abukhadra et al., 2024). Additionally, high proton activity suppresses metal ion adsorption by reducing the availability of negatively charged functional groups required for electrostatic attraction and surface complexation. These findings are consistent with previous reports on reduced metal ion uptake in strongly acidic environments due to proton shielding effects (Athman et al., 2019). As the pH increased from three to 5, a marked improvement in adsorption performance was observed, with adsorption capacities reaching 62.95 mg/g for Cd^2+^, 68.05 mg/g for Co^2+^, and 72.5 mg/g for Cu^2+^ at pH 5 (Figure 6A). This increase can be explained by: (A) reduction in proton interference, (B) development of negative surface charge, and (C) increased availability of surface binding sites. At moderate pH levels, fewer protons (H^+^) compete for adsorption sites, thereby allowing a greater number of metal ions to bind to the CTAB/MS surface. Also the progressive deprotonation of hydroxyl (-OH) groups at this pH level enhances the negative charge density on the serpentine nanoparticles, strengthening electrostatic attraction between the negatively charged adsorbent and the positively charged metal ions. The formation of negatively charged hydroxyl (-OH) and oxy (-O^−^) functional groups facilitates stronger metal-ligand interactions, improving metal uptake efficiency (Athman et al., 2019; Zhang et al., 2022). The observed trends underscore the importance of electrostatic interactions as the dominant adsorption mechanism at pH values up to 5. The significant increase in adsorption performance at this stage suggests that electrostatic attraction plays a major role in metal ion removal, particularly in aqueous environments with moderate acidity.

The highest adsorption efficiencies were recorded at pH 6, with maximum adsorption capacities of 84.25 mg/g for Cd^2+^, 81.5 mg/g for Co^2+^, and 83.1 mg/g for Cu^2+^ (Figure 6A). This enhancement is attributed to several key factors including the surface deprotonation and increased active sites. At pH 6, a significant number of Si–OH groups undergo deprotonation, leading to the formation of negatively charged siloxane sites that act as high-affinity binding sites for metal cations (Zhang et al., 2020). Also, the uptake efficiency at this pH level might be induced by the formation of inner-sphere complexes. The availability of hydroxyl (-OH) and oxy (-O^-^) groups facilitates chelation and inner-sphere complexation with metal ions, leading to stronger and more stable adsorption interactions (Zhang et al., 2020; Santoso et al., 2022). The reduction in the competitive effect of protons also essential factors affected the adsorption behavior. With fewer competing protons in the system, metal ions can more readily coordinate with functional groups, ensuring higher metal uptake. Furthermore, the reaction bight be enhanced by the operation of ligand exchange mechanisms; metal ions may replace adsorbed protons or other competing species, further stabilizing their attachment to the CTAB/MS surface (Athman et al., 2019; Santoso et al., 2022).

The optimal performance observed at pH 6 highlights the combined role of electrostatic attraction, surface complexation, and ligand exchange in achieving high metal removal efficiencies. These findings suggest that pH 6 is the most suitable condition for subsequent adsorption applications, as it maximizes adsorption site availability while minimizing competitive interference. The inspection of higher pH values beyond pH 7 was not investigated to avoid the impact of the precipitation of these metals as metal hydroxides.

#### 3.2.2 Influence of contact time

The influence of contact time on the adsorption efficiency of Cd^2+^, Co^2+^, and Cu^2+^ onto CTAB/MS was systematically examined over a time span ranging from 30 to 2,160 min (36 h) to determine the optimal duration required for equilibrium adsorption (Figure 6B). Throughout the experiment, key parameters were carefully controlled to eliminate external variations, ensuring that adsorption trends were solely attributed to the influence of contact time. These conditions included an initial metal ion concentration of 100 mg/L, pH 6, solution volume of 100 mL, temperature of 303 K (30°C), and a CTAB/MS dosage of 20 mg. The adsorption behavior exhibited a distinct two-phase trend. During the initial stage, a rapid increase in metal ion uptake was observed due to the high availability of active binding sites on the CTAB/MS surface (Santoso et al., 2022) (Figure 6B). This immediate uptake is characteristic of physisorption, where metal ions readily interact with negatively charged functional groups on the adsorbent (Athman et al., 2019; Santoso et al., 2022). As the adsorption process progressed, the rate of metal uptake gradually declined due to the progressive occupation of available active sites. With fewer unoccupied binding sites remaining, the competition among incoming metal ions intensified, reducing the overall rate of adsorption (El-Sherbeeny et al., 2021). This stage is largely governed by surface complexation mechanisms and diffusion limitations, where metal ions must penetrate deeper into the porous structure of CTAB/MS to access remaining active sites (Abdel Salam et al., 2022; Lin et al., 2021). After 960 min, the adsorption system approached equilibrium, with no further significant changes in the retention rates of Cd^2+^, Co^2+^, and Cu^2+^ (Figure 6B). The maximum adsorption capacities at this equilibrium state were determined to be 166 mg/g for Cd^2+^, 151.6 mg/g for Co^2+^, and 137.5 mg/g for Cu^2+^ (Figure 6B). The stabilization of adsorption capacity beyond this point indicates that the majority of active sites had been saturated, thereby restricting further adsorption (Santoso et al., 2022). The absence of significant adsorption beyond the equilibrium period suggests that: (A) the finite number of active sites on the CTAB/MS had been fully occupied; preventing additional metal ions from binding, (B) the rate of metal ion attachment equaled the rate of detachment, leading to a dynamic balance (Desorption–adsorption balance) in retention capacities (El-Sherbeeny et al., 2021; Tohdee et al., 2018), and (C) in later stages, adsorption may be restricted by intra-particle diffusion resistance, where metal ions struggle to penetrate deeper into the nanoporous matrix of CTAB/MS (Lin et al., 2021). Understanding the contact time dependence of metal ion adsorption is essential for optimizing industrial wastewater treatment processes, where efficiency and cost-effectiveness are critical considerations. The findings of this study highlight the rapid adsorption phase suggesting that CTAB/MS can effectively remove a substantial proportion of metal ions within the first few hours, making it suitable for adsorption systems in wastewater treatment plants.

#### 3.2.3 Influence of adsorbent dosage

The relationship between CTAB/MS dosage and metal ion removal efficiency was systematically evaluated, as depicted in Figure 6C, revealing a positive correlation between increasing adsorbent dosage and enhanced removal rates. The results demonstrated that as the dosage of CTAB/MS nanoparticles increased, the removal efficiency of Cd^2+^, Co^2+^, and Cu^2+^ significantly improved, ultimately reaching 92.2% for Cd^2+^, 90.1% for Co^2+^, and 87.2% for Cu^2+^ at an optimal dosage of 0.3 g. This substantial increase can be attributed to the higher availability of active adsorption sites and greater surface area provided by the increased concentration of CTAB/MS nanoparticles (Figure 6C). For Cd^2+^ ions, under the conditions of an initial metal concentration of 100 mg/L, pH 6, solution volume of 100 mL, and temperature of 303 K (30°C), the removal efficiency exhibited a steady increase with increasing CTAB/MS dosage. Specifically, Cd^2+^ removal rates improved from 18.3% at 0.02 g to 92.2% at 0.3 g, with intermediate efficiencies recorded at 35.9% (0.05 g), 53.4% (0.1 g), 70.6% (0.15 g), and 88.1% (0.2 g) (Figure 6C). A similar trend was observed for Co^2+^ and Cu^2+^, where removal efficiencies rose from 16.5% to 90.1% for Co^2+^ and from 15.5% to 87.2% for Cu^2+^ as the CTAB/MS dosage increased from 0.02 g to 0.3 g (Figure 6C). Understanding the dosage-dependent behavior of CTAB/MS in metal ion removal has several critical implications for environmental remediation and wastewater treatment applications. Therefore, determining the optimal dosage (0.3 g in this case) ensures cost-effectiveness and efficient resource utilization in large-scale applications. The ability of CTAB/MS to achieve over 90% removal efficiency for toxic metal ions at relatively low dosages highlights its potential as a viable adsorbent in industrial effluent treatment systems. The progressive enhancement in the metals removal efficiency with increasing CTAB/MS dosage is primarily attributed to three key factors. The first factor related to increased adsorption sites; a higher dosage of CTAB/MS nanoparticles results in a larger number of active binding sites, thereby improving the probability of metal ion interaction with the adsorbent surface (Salam et al., 2020; El-Soad et al., 2023). The second factor might be involved the enhancement in the surface area as more CTAB/MS is introduced into the system, the available surface area for adsorption expands, allowing more metal ions to be effectively captured and retained. The third factors might be assigned to the increment in the electrostatic interactions and surface complexation.

#### 3.2.4 Influence of starting concentrations

The impact of varying initial concentrations of Cd^2+^, Co^2+^, and Cu^2+^ ions on their adsorption by CTAB/MS was systematically examined over a concentration range of 50–350 mg/L (Figures 6D–F). To ensure experimental consistency and reproducibility, the adsorption studies were conducted under standardized conditions, including a CTAB/MS dosage of 0.02 g/L, a pH of 6, a contact time of 24 h, a solution volume of 100 mL, and temperatures ranging from 303 K to 323 K. As the initial concentrations of Cd^2+^, Co^2+^, and Cu^2+^ increased, the adsorption capacities of CTAB/MS also exhibited a corresponding rise, indicating that higher metal ion concentrations facilitate more efficient adsorbent-ions binding (Figures 6D–F). This behavior is primarily attributed to the increment in the ions diffusion and collision frequency. At higher concentrations, metal ions diffuse more readily through the solution and experience more frequent collisions with the active binding sites of CTAB/MS, thereby leading to an enhanced adsorption rate (Ahmed et al., 2024). While the adsorption capacity increased proportionally with initial metal ion concentration, a saturation threshold was observed beyond which additional increases in concentration had a negligible impact on adsorption. This suggests that the CTAB/MS active sites reached saturation, preventing further ion retention. The maximum adsorption capacities at different temperatures were determined for Cd^2+^ to be 307.65 mg/g at 303 K, 289.6 mg/g at 313 K, and 273.2 mg/g at 323 K (Figure 6D). For Co^2+^, the determined values were 300.15 mg/g at 303 K, 284.65 mg/g at 313 K, and 269.15 mg/g at 323 K (Figure 6E). For Cu^2+^, the measured values were 289.15 mg/g at 303 K, 268.2 mg/g at 313 K, and 254.65 mg/g at 323 K (Figure 6F). These findings underscore the temperature-dependent nature of adsorption, where higher temperatures (323 K) resulted in slightly lower adsorption capacities. This suggests that the adsorption process is partially exothermic, meaning that increasing temperature may reduce the affinity between metal ions and CTAB/MS, leading to lower adsorption capacities at elevated temperatures. Also, the findings highlight the potential of CTAB/MS as an effective adsorbent for metal ion sequestration from contaminated water, particularly in high-concentration effluents from industries such as mining, electroplating, and chemical manufacturing.

#### 3.2.5 Kinetic studies

##### 3.2.5.1 Intra-particle diffusion behavior

**FIGURE 7:**
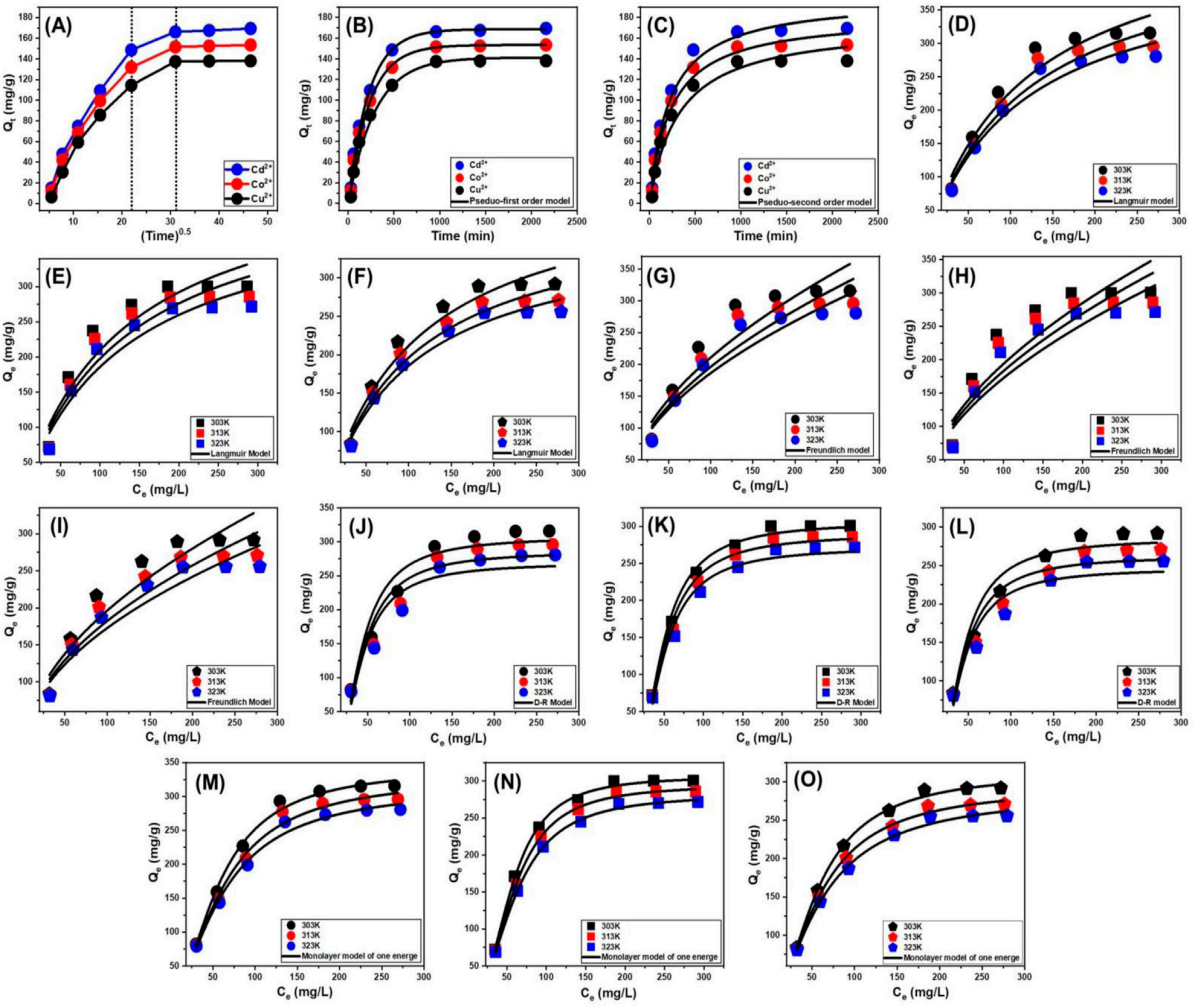
Fitting of the experimental results with the different kinetic and isotherm models including Intra-particle diffusion model **(A)**, Pseudo-first order kinetic model **(B)**, classic Langmuir model **(D–F)**, classic Freundlich model **(G–I)**, classic D-R model **(J–L)**, and advanced Monolayer model of one energy site **(M–O)**.

The intra-particle diffusion analysis provides insights into the adsorption mechanism of Cd^2+^, Co^2+^, and Cu^2+^ ions onto CTAB/MS, revealing a three-stage process characterized by distinct slopes. The observed adsorption curves did not exhibit clear intersections with the initial data points, suggesting that multiple adsorption mechanisms operate concurrently alongside ion diffusion through the CTAB/MS structure (Figure 7A). These findings imply that adsorption is not governed solely by a single diffusion process but rather follows a more complex multistage kinetic pathway (Ahmed et al., 2024; El Qada, 2020). The adsorption process can be categorized into three key phases suggesting that both surface adsorption and diffusion-controlled processes contribute to the removal of Cd^2+^, Co^2+^, and Cu^2+^ ions, indicating the complex nature of adsorption kinetics. The three suggested phase involved.

• External surface adsorption: During the initial phase, metal ions adhere to receptor sites located on the outer surface of the CTAB/MS adsorbent. This stage is primarily influenced by the availability of active adsorption sites and the electrostatic attraction between the positively charged metal ions and the negatively charged CTAB/MS surface (Figure 7A) (Lin et al., 2021).
• Intra-particle diffusion: As adsorption progresses, metal ions migrate into the internal porous structure of the CTAB/MS, where they interact with deeper active sites through diffusion-controlled processes (Lin et al., 2021; Neolaka et al., 2018). The rate of adsorption during this stage is primarily dependent on pore size, ion mobility, and surface charge interactions.
• Equilibrium and saturation: In the final phase, the adsorption process reaches equilibrium as active sites become fully occupied, and additional ion retention is minimal (Figure 7A). At this stage, thick layers of adsorbed metal ions form on the CTAB/MS surface, preventing further adsorption (Sayed et al., 2022; Salam et al., 2020).

##### 3.2.5.2 Kinetic modeling

To evaluate the adsorption kinetics of Cd^2+^, Co^2+^, and Cu^2+^ ions onto CTAB/MS, both the pseudo-first-order (P-F) (Figure 7B) and pseudo-second-order (P-S) (Figure 7C) models were employed. The nonlinear fitting approach was used to determine which model best describes the adsorption mechanism, with results assessed based on correlation coefficient (*R* ^2^) values and Chi-squared (χ^2^) values (Figures 7B,C; Table 1). The pseudo-first-order model exhibited a better fit, as indicated by higher *R* ^2^ values and lower χ^2^ values, suggesting that the adsorption process is predominantly governed by physisorption mechanisms, such as electrostatic attraction (Sherlala et al., 2019; Huang et al., 2018). This conclusion is further reinforced by the close alignment between experimental and theoretical adsorption capacities, which were found to be 168.7 mg/g for Cd^2+^, 153.4 mg/g for Co^2+^, and 141.2 mg/g for Cu^2+^ (Table 1). Although the pseudo-first-order model provided the best fit, the pseudo-second-order model also showed a reasonable correlation, indicating that chemisorption mechanisms—including electron exchange, surface complexation and internal diffusion—may also contribute to the adsorption process (Salam et al., 2020; Sherlala et al., 2019). The findings underscore the dual nature of adsorption, where physisorbed ions may cover layers of chemisorbed ions, resulting in high retention capacities. These insights are valuable for designing efficient adsorption systems for heavy metal removal from contaminated water sources (Jasper et al., 2020).

**TABLE 1:** The estimated parameters of the studied kinetic models.

	Model	Parameters	Values
Cd^2+^	Pseudo-First-order	*K*_1_ (1/min)	0.0044
		Qe _(Cal)_ (mg/g)	168.7
		*R* ^2^	0.98
		χ^2^	0.71
	Pseudo-Second-order	*k*_2_ (mg/g min)	2.2 × 10^−5^
		Qe _(Cal)_ (mg/g)	199.1
		*R* ^2^	0.97
		χ^2^	1.4
Co^2+^	Pseudo-First-order	*K*_1_ (1/min (	0.0043
		Qe _(Cal)_ (mg/g)	153.4
		*R* ^2^	0.98
		χ^2^	0.88
	Pseudo-Second-order	*k*_2_ (mg/g min)	2.33 × 10^−5^
		Qe _(Cal)_ (mg/g)	182.3
		*R* ^2^	0.97
		χ^2^	1.5
Cu^2+^	Pseudo-First-order	*K*_1_ (1/min (	0.003
		Qe _(Cal)_ (mg/g)	141.2
		*R* ^2^	0.96
		χ^2^	2.06
	Pseudo-Second-order	*k*_2_ (mg/g min)	1.76 × 10^−5^
		Qe _(Cal)_ (mg/g)	174.2
		*R* ^2^	0.95
		χ^2^	3.09

#### 3.2.6 Classic isotherm studies

The retention behavior of Cd^2+^, Co^2+^, and Cu^2+^ ions onto CTAB/MS particles was analyzed using three classical adsorption isotherm models: the Langmuir (Figures 7D–F), Freundlich (Figures 7G–I), and Dubinin-Radushkevich (D-R) (Figures 7J–L) models. The suitability of these models was assessed by evaluating the correlation coefficient (*R* ^2^) and Chi-squared (χ^2^) values (Table. 2). The results demonstrated that the Langmuir model provided the best fit, as indicated by its higher *R* ^2^ values and lower χ^2^ values, suggesting that adsorption occurred uniformly across the CTAB/MS surface (Huang et al., 2018; Dawodu et al., 2012). The Langmuir isotherm model assumes that adsorption occurs as a monolayer on a surface with a finite number of equivalent adsorption sites, where each site binds only one ion, and no interactions occur between adsorbed species (Jasper et al., 2020; Dawodu et al., 2012). The high correlation of experimental data with the Langmuir model indicates that adsorption onto CTAB/MS follows this monolayer coverage assumption, confirming the homogeneous distribution of active sites. The maximum adsorption capacities (Q_max_) were estimated as theoretical parameter of Langmuir model to be 491.9 mg/g at 303 K, 459.6 mg/g at 313 K, and 426.5 mg/g at 323 K for Cd^2+^ (Table. 2). For Co^2+^, the computed values were 481.8 mg/g at 303 K, 458.6 mg/g at 313 K, and 431.6 mg/g at 323 K (Table. 2). While for Cu^2+^ the values 434.3 mg/g at 303 K, 393.4 mg/g at 313 K, and 366.6 mg/g at 323 K (Table. 2). The adsorption capacity trend followed the order: Cd^2+^ > Co^2+^ > Cu^2+^, which suggests that Cd^2+^ ions exhibited the highest affinity toward CTAB/MS, likely due to differences in ionic radius, hydration energy, and surface interactions.

**TABLE 2:** The estimated parameters of the classic isotherm models.

	Models	Parameters	303 K	313 K	323 K
Cd^2+^	Langmuir	*Q*_max_ (mg/g)	491.9	459.6	426.5
		*b*(L/mg)	0.0088	0.0085	0.0084
		*R* ^2^	0.95	0.96	0.966
		χ^2^	2.52	1.99	1.51
	Freundlich	1/*n*	0.543	0.54	0.52
		*k*_*F*_ (mg/g)	17.2	16.5	16.3
		*R* ^2^	0.88	0.89	0.9
		χ^2^	6.1	5	4.2
	D-R model	β (mol^2^/KJ^2^)	0.57	0.58	0.59
		*Q*_*m*_ (mg/g)	307.6	285.8	269.2
		*R* ^2^	0.94	0.92	0.91
		χ^2^	3.01	3.8	3.9
		*E* (KJ/mol)	0.93	0.924	0.92
Co^2+^	Langmuir	*Q*_max_ (mg/g)	481.8	458.6	431.6
		*b*(L/mg)	0.0076	0.0075	0.0075
		*R* ^2^	0.9	0.91	0.92
		χ^2^	5.5	4.6	3.5
	Freundlich	1/*n*	0.555	0.554	0.552
		*k*_*F*_ (mg/g)	15	14.2	13.6
		*R* ^2^	0.82	0.83	0.85
		χ^2^	10	8.8	7.1
	D-R model	β (mol^2^/KJ^2^)	0.76	0.78	0.79
		*Q*_*m*_ (mg/g)	305.9	289.6	271.8
		*R* ^2^	0.99	0.98	0.98
		χ^2^	0.3	0.5	0.8
		*E* (KJ/mol)	0.81	0.8	0.79
Cu^2+^	Langmuir	*Q*_max_ (mg/g)	434.3	393.4	366.9
		*b*(L/mg)	0.0095	0.0094	0.01
		*R* ^2^	0.96	0.969	0.97
		χ^2^	1.7	1.1	0.8
	Freundlich	1/*n*	0.51	0.5	0.48
		*k*_*F*_ (mg/g)	18.66	18.62	18.59
		*R* ^2^	0.89	0.9	0.91
		χ^2^	4.7	3.6	2.8
	D-R model	β (mol^2^/KJ^2^)	0.58	0.57	0.55
		*Q*_*m*_ (mg/g)	285	262.2	246.2
		*R* ^2^	0.95	0.94	0.93
		χ^2^	1.8	1.9	2.3
		*E* (KJ/mol)	0.92	0.93	0.95

The D-R isotherm model was used to assess the heterogeneity of adsorption sites on the CTAB/MS surface, providing insights into the adsorption energy and mechanism (Kusuma et al., 2024). The adsorption energy (E) parameter derived from the D-R model helps distinguish between physical and chemical adsorption mechanisms. E < 8 kJ/mol suggests physical adsorption (physisorption), 8 kJ/mol ≤ E ≤ 16 kJ/mol reflects weak chemical adsorption or mixed mechanisms, and E > 16 kJ/mol demonstrates strong chemical adsorption (chemisorption) (Kusuma et al., 2024; Ahmed et al., 2024). The E values for Cd^2+^, Co^2+^, and Cu^2+^ adsorption onto CTAB/MS were all below 8 kJ/mol, indicating that the adsorption process was predominantly governed by physical interactions (Table. 2). This suggests that electrostatic forces, van der Waals interactions, and weak hydrogen bonding played a crucial role in metal ion retention, rather than strong covalent bonding or ion exchange mechanisms. Since adsorption occurs primarily *via* physisorption, regeneration and reuse of CTAB/MS could be feasible through simple desorption techniques. This also implies that CTAB/MS does not undergo significant structural changes, ensuring stability and reusability over multiple adsorption cycles. These findings suggest that CTAB/MS is an effective, stable, and reusable adsorbent for removing heavy metal contaminants, making it a promising material for industrial and environmental remediation applications.

#### 3.2.7 Advanced isotherm modeling

Recent developments in isotherm modeling, based on statistical physics theory, have been applied to evaluate the adsorption of Cd^2+^, Co^2+^, and Cu^2+^ ions onto CTAB/MS surfaces. These advanced models offer a comprehensive understanding of adsorption behavior by integrating steric and energetic parameters. Steric parameters include the number of adsorbed ions per site (n), the density of occupied sites (Nm), and the saturation adsorption capacity (Q_sat_). Energetic parameters encompass adsorption energy (ΔE), internal energy (E_int_), free enthalpy (G), and entropy (Sa). Experimental data were analyzed using nonlinear isotherm equations, with the Levenberg–Marquardt algorithm employed for multivariable nonlinear regression. The findings revealed that Cd^2+^, Co^2+^, and Cu^2+^ adsorption on CTAB/MS is best described by a monolayer adsorption model involving a single energy site (Figures 7M–O; Table. 3).

**TABLE 3:** The estimated parameters of the advanced monolayer isotherm model of one energy site.

		303K	313 K	323 K
Cd^2+^	*R* ^2^	0.995	0.989	0.98
	*χ* ^2^	0.27	0.53	0.46
	*n*	1.83	1.69	1.61
	*N*_m_ (mg/g)	187	195	195.7
	*Q*_sat_ (mg/g)	336.6	329.5	315
	*C*_1/2_ (mg/L)	57.4	61.3	62
	Δ*E* (kJ/mol)	−11.89	−12.07	−12.13
Co^2+^	*R* ^2^	0.998	0.999	0.999
	*χ* ^2^	0.093	0.04	0.04
	*n*	2.5	2.4	2.2
	*N*_m_ (mg/g)	120.2	122.5	126
	*Q*_sat_ (mg/g)	300.5	294	277.2
	*C*_1/2_ (mg/L)	55.2	57.5	59.3
	Δ*E* (kJ/mol)	−12.04	−12.36	−12.66
Cu^2+^	*R* ^2^	0.998	0.997	0.995
	*χ* ^2^	0.085	0.094	0.15
	*n*	1.82	1.69	1.55
	*N*_m_ (mg/g)	172	173.1	183.4
	*Q*_sat_ (mg/g)	313	292.5	284.2
	*C*_1/2_ (mg/L)	55.5	56.6	59
	Δ*E* (kJ/mol)	−5.8	−5.86	−5.98

##### 3.2.7.1 Steric parameters

###### 3.2.7.1.1 Number of adsorbed ions per site

**FIGURE 8:**
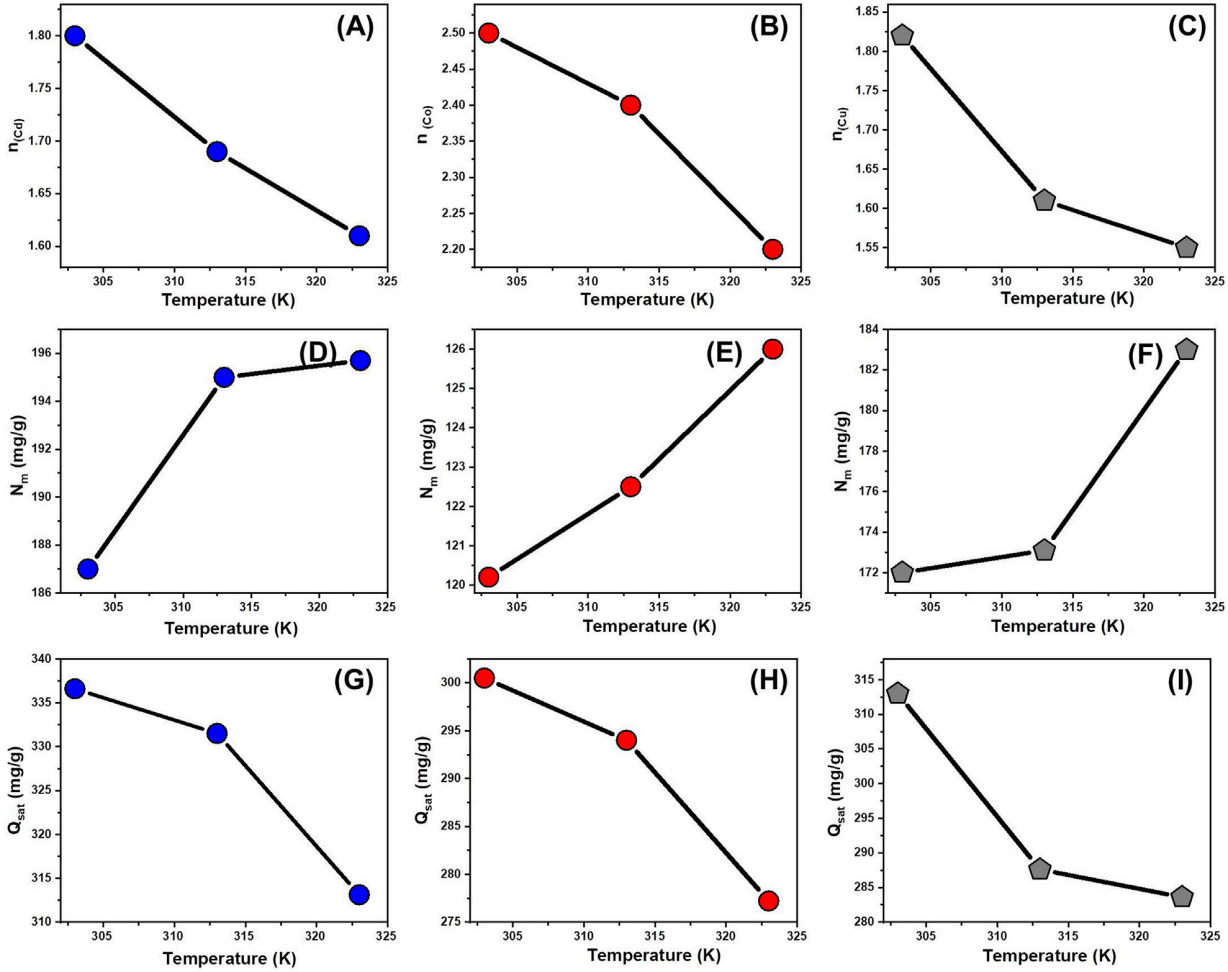
Changes in the steric parameters during the uptake of the metals at different operating temperature levels including number of adsorbed ions per site [Cd^2+^ **(A)**, Co^2+^ **(B)**, and Cu^2+^ **(C)**], active sites density [Cd^2+^ **(D)**, Co^2+^ **(E)**, and Cu^2+^ **(F)**], and saturation uptake capacity [Cd^2+^ **(G)**, Co^2+^ **(H)**, and Cu^2+^ **(I)**].

The number of adsorbed ions per site (n) provides insight into the orientation and interaction mechanisms of Cd^2+^, Co^2+^, and Cu^2+^ ions on CTAB/MS surfaces. If n < 1, adsorption follows a horizontal orientation, meaning multiple ions occupy a single active site, forming multi-ionic interactions (Sayed et al., 2022; Mobarak et al., 2021). For Cd^2+^, the n values ranged between 1.61 and 1.8. For Co^2+^, the n values varied between 2.2 and 2.5. For Cu^2+^, the n values ranged from 1.5 to 1.82 (Figures 8A–C; Table 3). Since all values exceed 1, this suggests that Cd^2+^, Co^2+^, and Cu^2+^ ions exhibit multi-ionic interactions during adsorption. Each adsorption site can hold up to two Cd^2+^ ions and up to three Co^2+^ or Cu^2+^ ions, with ions aligning vertically rather than in a parallel arrangement. The n values decrease with rising temperature, suggesting that higher temperatures reduce aggregation behavior during adsorption (Mobarak et al., 2021; Dhaouadi et al., 2021).

###### 3.2.7.1.2 Occupied active sites density

The Nm parameter represents the density of adsorption sites on the CTAB/MS surface, providing insight into the total number of binding sites available for Cd^2+^, Co^2+^, and Cu^2+^ ions (Table 3). Cd^2+^ adsorption site densities (N_m_) were 187 mg/g at 303 K, 195 mg/g at 313 K, and 195.7 mg/g at 323 K (Figure 8D)For the Co^2+^ adsorption site densities (Nm) were 120.2 mg/g at 303 K, 122.5 mg/g at 313 K, 126 mg/g at 323 K (Figure 8E). For the computed, Cu^2+^ adsorption site densities, the estimated values were 172 mg/g at 303 K, 173.1 mg/g at 313 K, and 183 mg/g at 323 K (Figure 8F). However, as temperature rises, the number of filled adsorption sites decreases. This is likely due to increased aggregation of ions, leading to reduced available sites (Sellaoui et al., 2020; Dhaouadi et al., 2020).

###### 3.2.7.1.3 Saturation adsorption capacity

The saturation adsorption capacity (Q_sat_) represents the maximum potential for metal ion adsorption onto CTAB/MS. Its value is influenced by both N_m_ (density of occupied sites) and n (number of ions per site). The estimated Cd^2+^ adsorption capacities (Q_sat_) were 336.6 mg/g at 303 K, 329.5 mg/g at 313 K, and 315 mg/g at 323 K (Figure 8G; Table 3). For the Co^2+^ adsorption capacities, the computed values were 300.5 mg/g at 303 K, 294 mg/g at 313 K, and 277.2 mg/g at 323 K (Figure 8H; Table 3). The estimated capacities for Cu^2+^ were 313 mg/g at 303 K, 287.6 mg/g at 313 K, and 283.6 mg/g at 323 K (Figure 8I; Table 3). The gradual decrease in Q_sat_ with increasing temperature suggests that the adsorption process is exothermic. Therefore, as temperature rises, the thermal motion of metal ions increases, leading to reduced binding efficiency and lower adsorption capacity. The exothermic nature of adsorption aligns with the observation that higher temperatures promote ion collisions, reducing the retention of Cd^2+^, Co^2+^, and Cu^2+^ ions (Sellaoui et al., 2020; Dhaouadi et al., 2020). The temperature-dependent variation in Q_sat_ is more closely linked to changes in Nm (occupied site density) rather than n (ions per site). This suggests that the number and availability of active sites play a more critical role in determining adsorption efficiency than the adsorption capacity of individual sites. The marked selectivity of CTAB/MS to the different metal ions (Cd^2+^ > Co^2+^ > Cu^2+^) can be attributed to several physicochemical factors, including ionic radius, hydration energy, and electrostatic interactions with the CTAB-functionalized magnesium silicate surface. Cadmium ions (Cd^2+^), with a relatively larger ionic radius (∼0.97 Å) and lower hydration energy, exhibit less hydration shell resistance and can more easily approach and interact with the negatively charged functional groups on the adsorbent surface (Akinola et al., 2025). In contrast, Co^2+^ and Cu^2+^ ions have smaller radii (∼0.74 Å and ∼0.73 Å, respectively) and stronger hydration shells, which may reduce their diffusion rate and accessibility to binding sites (Ibezim-Ezeani et al., 2012). Moreover, the difference in electronic configurations and preferences for complexation may also contribute to the adsorption behavior, where Cd^2+^ tends to form more labile surface complexes (Palazzetti, 2025). These factors collectively explain the higher affinity of the CTAB/MS adsorbent toward Cd^2+^ ions.

##### 3.2.7.2 Adsorption energy

The adsorption energy (ΔE) plays a crucial role in identifying the fundamental mechanisms governing the adsorption of Cd^2+^, Co^2+^, and Cu^2+^ ions on the CTAB/MS surface. It allows for differentiation between physical and chemical adsorption processes. Generally, chemical adsorption is characterized by energy values exceeding 80 kJ/mol, whereas physical adsorption involves lower energy values, typically 40 kJ/mol or less. Within physical adsorption, specific interaction types can be categorized based on their energy ranges: coordination exchange (∼40 kJ/mol), hydrogen bonding (<30 kJ/mol), dipole interactions (2–29 kJ/mol), van der Waals forces (4–10 kJ/mol), and hydrophobic bonds (∼5 kJ/mol) (Sellaoui et al., 2016; Foo and Hameed, 2009).

The adsorption energy values for Cd^2+^, Co^2+^, and Cu^2+^ were calculated using Equation (8), which incorporates key thermodynamic parameters, including the solubility of cadmium in water (S), gas constant (R = 0.008314 kJ/molK), absolute temperature (T), and ion concentration at half-saturation. The obtained energy ranges were up to −12.13 kJ/mol for Cd^2+^, up to −12.66 kJ/mol for Co^2+^, and up to −5.98 kJ/mol for Cu^2+^ (Table 3). These results strongly indicate that the adsorption of these metal ions onto CTAB/MS is predominantly driven by physical adsorption mechanisms, including van der Waals forces, hydrophobic interactions, dipole interactions, and hydrogen bonding. The consistently negative ΔE values further validate that the adsorption process is exothermic, aligning with previous experimental findings.

##### 3.2.7.3 Thermodynamic functions

###### 3.2.7.3.1 Entropy

**FIGURE 9:**
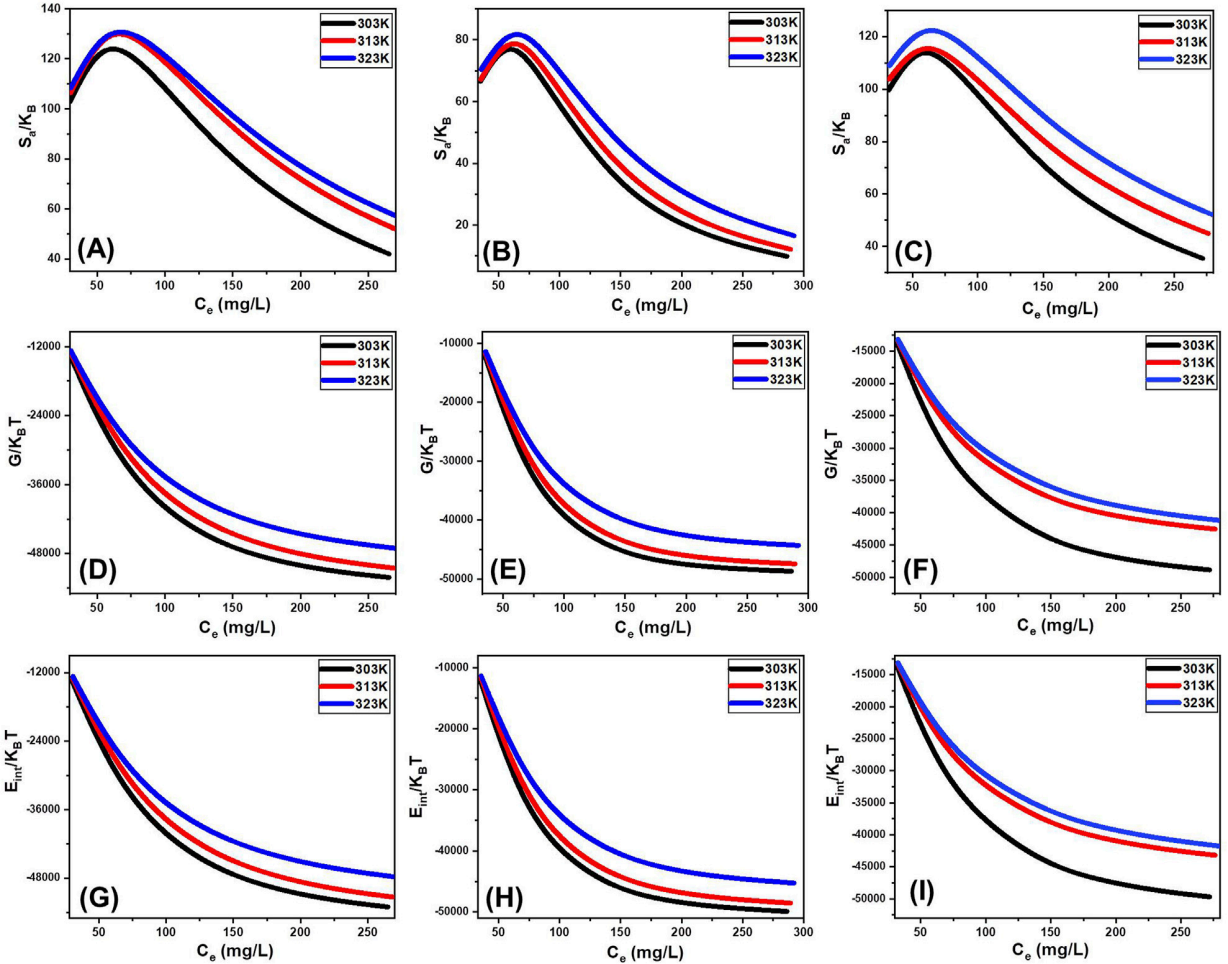
Changes in the thermodynamic functions during the uptake of the metals at different operating temperature levels including entropy [Cd^2+^ **(A)**, Co^2+^ **(B)**, and Cu^2+^ **(C)**], enthalpy [Cd^2+^ **(D)**, Co^2+^ **(E)**, and Cu^2+^ **(F)**], and internal energy [Cd^2+^ **(G)**, Co^2+^ **(H)**, and Cu^2+^ **(I)**].

Entropy (Sa) provides insight into the order and disorder changes occurring on the CTAB/MS surface during the adsorption process of Cd^2+^, Co^2+^, and Cu^2+^ ions. A thorough analysis of Sa (Equation 9), guided by parameters such as density of occupied adsorption sites (Nm), the number of ions per site (n), and ion concentration at half-saturation (C_1/2_), revealed a slight decline in entropy with increased ion adsorption, particularly at higher ion concentrations (Figures 9A–C). This decline signifies a reduction in surface disorder, suggesting that as more adsorption sites become occupied, the material’s structural mobility decreases (Ahmed et al., 2024). This entropy behavior supports the spontaneous nature of adsorption, where ions become increasingly restricted as they bind to the CTAB/MS surface. The decreasing entropy trend highlights the adsorbent’s efficiency in capturing metal ions, particularly under conditions of higher initial ion concentrations (Dhaouadi et al., 2020). Experimental data demonstrated that Cd^2+^ exhibited the highest entropy values, measuring 129.4 mg/L at 303 K, 134.8 mg/L at 313 K, and 135.3 mg/L at 323 K (Figure 9A). Comparatively, the entropy values for Co^2+^ were 82.7 mg/g at 303 K, 84.4 mg/g at 313 K, and 87 mg/g at 323 K (Figure 9B), while for Cu^2+^, they were 119.1 mg/g at 303 K, 119.9 mg/g at 313 K, and 126.8 mg/g at 323 K (Figure 9C). These equilibrium values align well with the predicted concentrations required for half-saturation of the silica nanostructures, reinforcing the notion that as adsorption progresses, fewer binding sites remain available (Dhaouadi et al., 2020).

###### 3.2.7.3.2 Internal energy and free enthalpy

The internal energy (E_int_) and free enthalpy (G) associated with the adsorption of Cd^2+^, Co^2+^, and Cu^2+^ ions onto CTAB/MS were assessed using Equations (8) and (9). These calculations incorporated key adsorption parameters, including the ion concentration at half-saturation (C_1/2_), translation partition function (Zv), occupied adsorption site density (Nm), and the number of ions per site (n). The results indicated that the free enthalpy values (G) were consistently negative, demonstrating that the adsorption process is both spontaneous and exothermic (Sellaoui et al., 2016) (Figures 9D–F). Notably, G values decreased as temperature increased from 303 K to 313 K, as illustrated in Figures 9D–F. This inverse relationship between G and temperature underscores the exothermic nature of the adsorption reaction, affirming that lower temperatures enhance metal ion retention. Furthermore, the negative free enthalpy (G) values confirm that the adsorption is thermodynamically favorable across all studied temperatures (Sellaoui et al., 2016; Foo and Hameed, 2009). Similarly, the internal energy (E_int_) values were also negative and displayed a progressive reduction as temperature increased, further reinforcing the exothermic behavior of the adsorption process (Figures 9G–I). The decline in E_int_ with increasing temperature aligns with the experimental data indicating weaker interactions at elevated temperatures, which has practical relevance for designing adsorption-based filtration systems operating at ambient or controlled temperatures. The spontaneous nature of adsorption suggests that no external energy input is required, making CTAB/MS a cost-effective and efficient adsorbent for industrial applications.

#### 3.2.8 Recyclability

The long-term applicability of the CTAB-functionalized magnesium silicate (CTAB/MS) adsorbent was thoroughly evaluated through a five-cycle regeneration study, aiming to assess its reusability, sustainability, and potential environmental impact. Regeneration was performed using a simple and energy-efficient procedure: adsorbents were washed with distilled water at 50 °C for 60 min to remove residual metal ions and then dried at 60 °C for 1 hour. Adsorption experiments were consistently repeated under optimized conditions (metal ion concentration: 100 mg/L; adsorbent dosage: 0.02 g/L; pH: 6; 100 mL solution volume; 30 °C; 16-h contact time).

**FIGURE 10:**
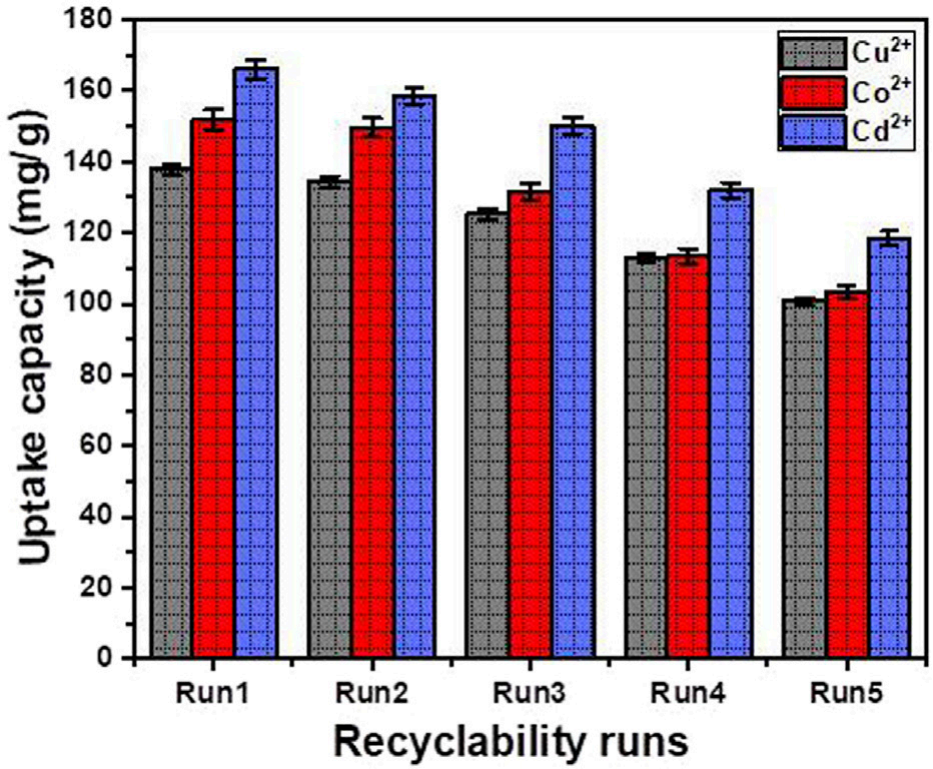
The recyclability properties of CTAB/MS structures as adsorbent for Cd^2+^, Co^2+^, and Cu^2+^.

The results demonstrated notable stability in adsorption efficiency across successive cycles (Figure 10). For Cd^2+^, the uptake decreased moderately from 165.95 mg/g in the first cycle to 118.55 mg/g by the fifth. Co^2+^ and Cu^2+^ showed similar trends, with final cycle values of 103.2 mg/g and 100.65 mg/g, respectively. The gradual reduction can be attributed to partial surface saturation, irreversible complexation with surface-active sites, and minimal material loss during the regeneration process. Despite these reductions, CTAB/MS maintained over 70% of its initial performance, affirming its robustness as a reusable, cost-effective, and eco-friendly adsorbent for heavy metal remediation.

Importantly, beyond its use as an adsorbent, the spent CTAB/MS material holds promising potential for repurposing in environmental and catalytic applications. The immobilized metal ions (Cd^2+^, Co^2+^, and Cu^2+^) could act as active catalytic centers when thermally treated or chemically processed, potentially enabling the transformation of the metal-laden adsorbent into a metal-magnesium silicate composite catalyst. Such structures may be applicable in oxidation reactions, degradation of organic pollutants, or photocatalytic systems. Furthermore, the recovery of adsorbed heavy metals from the exhausted adsorbent could add significant value and support resource sustainability. Metal desorption using acid leaching, chelation, or electrochemical processes could allow for partial or complete retrieval of the bound metals, reducing secondary waste and enabling potential metal recycling. These strategies align with sustainable circular economy principles, enhancing both economic feasibility and environmental safety. In cases where reuse or regeneration is not viable, safe management of the spent adsorbent is essential. Immobilizing the material in cementitious matrices or using it in geopolymer formulations could mitigate leaching risks while contributing to sustainable construction practices. These approaches align with global best practices, as emphasized in recent work on tailored recycling strategies and sustainable reuse of spent adsorbents (Faheem et al., 2024) (Faheem et al., 2024). Overall, the CTAB/MS adsorbent not only exhibits excellent regeneration capability but also offers versatile post-use pathways that promote resource recovery, minimize environmental risks, and open up new opportunities in catalytic and environmental fields.

#### 3.2.9 Comparison study

The adsorption efficiency of CTAB/MS was assessed in comparison with other adsorbents documented in the literature, revealing its better capability for Cd^2+^, Co^2+^, and Cu^2+^ removal (Supplementary Table S2). CTAB/MS outperformed several conventional carbon-based adsorbents including activated carbon and graphene oxide composites in addition to clay-based adsorbents, including various forms of montmorillonite such as, as well as composite materials like SiO_2_/kaolinite/Fe_2_O_3_ and Nano-Kaolinite. In comparison with zeolite-based adsorbents, CTAB/MS exhibited higher adsorption capacities than several forms of zeolite based structures and composites. Likewise, when compared to chitosan-based adsorbents such as chitosan-magnetic nanocomposites and chitosan/clay composites or more advanced adsorbents like MWCNT/IO composites, Na_2_Ti_2_O_5_-NTs, H_2_Ti_2_O_5_-NTs, K_2_Ti_6_O_13_, and mesoporous silica-based materials CTAB/MS demonstrated a stronger ability to capture and retain heavy metal ions. Finally, when evaluated against bulk serpentine and CTAB/MS still demonstrated a promising potential for metal ion removal, further emphasizing its effectiveness as an adsorbent (Supplementary Table S2). The superior performance of CTAB/MS can be attributed to its unique structural properties, including enhanced surface reactivity, expanded interlayer spacing, and increased density of active binding sites. These characteristics enable efficient adsorption through multiple mechanisms, including ion-exchange, electrostatic interactions, and surface complexation, making CTAB/MS a strong candidate for practical heavy metal remediation applications.

## 4 Conclusions

This study successfully synthesized and optimized CTAB-functionalized magnesium silicate (CTAB/MS) nanotubes as a highly efficient and recyclable adsorbent for the removal of Cd^2+^, Co^2+^, and Cu^2+^ from contaminated water. The adsorption process was strongly pH-dependent, with maximum uptake at pH 6. The kinetic analysis confirmed a pseudo-first-order model, suggesting rapid initial metal ion retention due to surface adsorption, followed by intra-particle diffusion into mesopores. The adsorption isotherm followed the Langmuir model, indicating monolayer adsorption on homogeneously distributed active sites, with maximum adsorption capacities of 491.9 mg/g (Cd^2+^), 481.8 mg/g (Co^2+^), and 434.3 mg/g (Cu^2+^) at 303 K, surpassing many conventional adsorbents. Thermodynamic studies revealed that adsorption was exothermic and spontaneous, with negative free enthalpy (G) and internal energy (E_int_) values, further confirming the physical nature of the retention mechanism. The adsorption energy (ΔE) values remained below 12.66 kJ/mol, reinforce the weak physical interactions such as hydrogen bonding, van der Waals forces, and dipole interactions as the primary driving forces. Statistical physics-based modeling further demonstrated the influence of steric factors, such as the density of occupied adsorption sites (Nm) and the number of ions retained per site (n), which influenced adsorption efficiency. Reusability tests demonstrated that CTAB/MS retained over 70% of its adsorption efficiency after five consecutive regeneration cycles, proving its long-term stability and industrial feasibility. Compared to other adsorbents, including clays, zeolites, carbon materials, and mesoporous silica, CTAB/MS exhibited better adsorption capacity and improved recyclability, making it a promising candidate for large-scale wastewater treatment applications. CTAB/MS offer a cost-effective and sustainable solution for heavy metal removal in industrial wastewater treatment, mining effluent processing, and water purification systems.

## 5 Recommendation

Further studies should focus on scaling up the synthesis process, evaluating long-term stability in real-world wastewater environments, and integrating CTAB/MS with hybrid treatment systems to enhance its performance and operational feasibility.

## Data Availability

The original contributions presented in the study are included in the article/Supplementary Material, further inquiries can be directed to the corresponding author.
