# Supplementary material for: Engineering high-performance CTAB-functionalized magnesium silicate nano-adsorbent for efficient removal of Cd2+, Co2+, and Cu2+ from single-metal aqueous solutions
Source: Front Chem. 2025 May 8;13:1583305. doi: 10.3389/fchem.2025.1583305 (PMC12095273; doi:10.3389/fchem.2025.1583305)
Supplement: Supplementary file 1 [file DataSheet1.docx]

Table.S1. Nonlinear equations of kinetic, classic isotherm, and advanced isotherm models

| Kinetic models | | |
| --- | --- | --- |
| Model | Equation | Parameters |
| Pseudo-first-order | $Q_{t}=Q_{e} (1-e^{{-k}_{1}.t})$ | Q_t_ (mg/g) is the adsorbed ions at time (t), and K_1_ is the rate constant of the first-order adsorption (1/min) |
| Pseudo-second-order | $Q_{t}=\frac{Q_{e}^{2}k_{2}t}{1+Q_{e}k_{2}t}$ | Qe is the quantity of adsorbed ions after equilibration (mg/g), and K_2_ is the model rate constant (g/mg min). |
| Classic Isotherm models | | |
| Model | Equation | Parameters |
| Langmuir | $Q_{e}=\frac{Q_{max} bC_{e}}{(1+bC_{e})}$ | *C_e_* is the rest ions concentrations (mg/L), *Q_max_* is the theoritical maximum adsorption capacity (mg/g), and *b* is the Langmuir constant (L/mg) |
| Freundlich | $Q_{e}=K_{f}C_{e}^{1/n}$ | K_F_ (mg/g) is the constant of Freundlich model related to the adsorption capacity and n is the constant of Freundlich model related to the adsorption intensities |
| Dubinin–Radushkevich | $Q_{e}=Q_{m}e^{-\betaɛ^{2}}$ | β (mol^2^/KJ^2^) is the D-R constant, ɛ (KJ^2^/mol^2^) is the Polanyi potential, and Q_m_ is the adsorption capacity (mg/g) |
| Advanced isotherm models | | |
| Model | Equation | Parameters |
| Monolayer model with one energy site (Model 1) | $Q=nN_{o} =\frac{nN_{M}}{1+{(\frac{C1/2}{C})}^{n}}=\frac{Q_{o}}{1+{(\frac{C1/2}{C})}^{n}}$ | Q is the adsorbed quantities in mg/g  n is the number of adsorbed ion per site  Nm is the density of the effective receptor sites (mg/g)  Q_o_ is the adsorption capacity at the saturation state in mg/g  C1/2 is the concentration of the ions at half saturation stage in mg/L  C1 and C2 are the concentrations of the ions at the half saturation stage for the first active sites and the second active sites, respectively  n1 and n2 are the adsorbed ions per site for the first active sites and the second active sites, respectively |
| Monolayer model with two energy sites (Model 2) | $Q=\frac{n_{1}N_{1M}}{1+{(\frac{C_{1}}{C})}^{n_{1}}}+\frac{n_{2}N_{2M}}{1+{(\frac{C_{2}}{C})}^{n_{2}}}$ |  |
| Double layer model with one energy site (Model 3) | $Q=Q_{o}\frac{({\frac{C}{C1/2})}^{n}+2({\frac{C}{C1/2})}^{2n}}{1+({\frac{C}{C1/2})}^{n}+({\frac{C}{C1/2})}^{2n}}$ |  |
| Double layer model with two energy sites (Model 3) | $Q=Q_{o}\frac{({\frac{C}{C1})}^{n}+2({\frac{C}{C2})}^{2n}}{1+({\frac{C}{C1})}^{n}+({\frac{C}{C2})}^{2n}}$ |  |

**Table.S2.** comparison between the adsorption performances of CTAB/MS and other studied adsorbents in literature

| Adsorbents | Heavy metals | Adsorption capacity (mg/g) | References |
| --- | --- | --- | --- |
| Na-Mtt  Na-Mtt  Na-Mtt | Cu (II) | 9.66 | Li et al., 2024  Li et al., 2024  Li et al., 2024 |
|  | Co (II) | 12.09 |  |
|  | Cd (II) | 22.95 |  |
| FAU-zeolites  FAU-zeolites | Cu (II) | 57.803 | Joseph et al., 2020  Joseph et al., 2020 |
|  | Cd (II) | 53.476 |  |
| SiO_2_/kaolinite/Fe_2_O_3_ composites | Cd (II) | 163.93 | Awwad et al., 2022 |
| Nano-Kaolinite | Cd (II) | 232.6 | Awwad et al., 2020 |
| Mt-Kaolinite/TiO2 | Cd (II) | 42.9 | [Djukic](https://www.researchgate.net/profile/Andjelka-Djukic-2?_sg%5B0%5D=hCSEPQmkD64g-TNktj6cGwX3pKsmbGUWrwZuhpOv8QqDpOftPwIr6-qm40w8G6HLTWgAFik.e2d64GijlslDxdSxnN63jEkVGVg1dzOPxAI6GSDMWn9kI_Mk2V1uPR01oK3kr7QTrqsPAiL8F3UjbFIJIYQewQ&_sg%5B1%5D=6sIRa1UalrrauO27z7sWcc4HwYyqRLpRppqaP5U-oINWOQKvyyfnzMBbzfzepBRJG3lxnEA.qfYaT5jcaBEmuf1U3B4yaeZlFmJlmcdtDKRCCnjRF9qYsd0joF4uAsF0e1kzTBE7rx_3gNtrhSV0D4nboRlbpw&_tp=eyJjb250ZXh0Ijp7ImZpcnN0UGFnZSI6ImhvbWUiLCJwYWdlIjoicHVibGljYXRpb24iLCJwb3NpdGlvbiI6InBhZ2VIZWFkZXIifX0) et al., 2014 |
| Kaolinite-hydro char (HCK) | Cd (II) | 63.19 | Al-Sawid et al., 2023 |
| Acrylic acid/bentonite | Cd (II) | 416.67 | Bulut et al., 2009 |
| Bentonite | Cu (II) | 558.36 | Chang et al., 2020  Chang et al., 2020 |
| Bentonite/GO | Cu (II) | 248.93 |  |
| Montmorillonite/chitosan gel | Cu (II) | 119.42 | Qin et al., 2020 |
| Acid-Kaolinite | Cu (II) | 42.01 | Tohdee et al., 2018 |
| Moroccan natural clay | Cu (II) | 48.24 | Barrak et al., 2022 |
| Tunisian natural clay | Cu (II) | 21.93 | [Khalfa](https://link.springer.com/article/10.1007/s13762-019-02614-x#auth-L_-Khalfa-Aff1) et al., 2023 |
| kaolin clay | Cu (II) | 52.63 | Bahah et al., 2019 |
| Montmorillonite (MMT) Egypt | Cu (II) | 7.6 | Gafoor et al., 2023 |
| Na2Ti2O5-NTs | Co (II) | 78.9 | [Mei Li](https://link.springer.com/article/10.1007/s41365-016-0135-1#auth-Dong_Mei-Li-Aff1) et al., 2016 |
| MWCNT/IO | Co (II) | 2.955 | Wang et al., 2011 |
| Chitosan-magnetic nanocomposite | Co (II) | 53.19 | Sayed et al., 2022 |
| bentonite | Co (II) | 9.97 | [Hashemian](https://www.researchgate.net/profile/Saeedeh-Hashemian?_tp=eyJjb250ZXh0Ijp7ImZpcnN0UGFnZSI6InB1YmxpY2F0aW9uIiwicGFnZSI6InB1YmxpY2F0aW9uIn19) et al., 2014  [Hashemian](https://www.researchgate.net/profile/Saeedeh-Hashemian?_tp=eyJjb250ZXh0Ijp7ImZpcnN0UGFnZSI6InB1YmxpY2F0aW9uIiwicGFnZSI6InB1YmxpY2F0aW9uIn19) et al., 2014  [Hashemian](https://www.researchgate.net/profile/Saeedeh-Hashemian?_tp=eyJjb250ZXh0Ijp7ImZpcnN0UGFnZSI6InB1YmxpY2F0aW9uIiwicGFnZSI6InB1YmxpY2F0aW9uIn19) et al., 2014 |
| Fe3O4 | Co (II) | 15.22 |  |
| Fe3O4-bentonite | Co (II) | 18.76 |  |
| Zeolitized diatomite | Cd (II) | 97.6 | Abukhadra et al., 2023  Abukhadra et al., 2023 |
| Cellulose/zeolite | Cd (II) | 117.2 |  |
| MnO-Kaolinite | Cd (II) | 36.4 | Sari et al., 2014 |
| Kaolinite | Cd (II) | 4.38 | [Unuabonah](https://link.springer.com/article/10.1007/s10450-008-9142-9#auth-E__I_-Unuabonah-Aff1) et al., 2008 |
| PVA-modified Kaolinite | Cd (II) | 29.8 | [Unuabonah](https://link.springer.com/article/10.1007/s10450-008-9142-9#auth-E__I_-Unuabonah-Aff1) et al., 2008 |
| Al-Pillared Mt | Cd (II) | 14.2 | Ma et al., 2015 |
| Chitosan/clay | Cd (II) | 72.31 | [Tirtom](https://www.researchgate.net/profile/Vedia-Nueket-Tirtom?_tp=eyJjb250ZXh0Ijp7ImZpcnN0UGFnZSI6InB1YmxpY2F0aW9uIiwicGFnZSI6InB1YmxpY2F0aW9uIn19) et al., 2012 |
| TiO_2_-kaolinite nanocomposite | Cd (II) | 250 | [Kubilay](https://link.springer.com/article/10.1007/s10450-007-9003-y#auth-__-Kubilay-Aff1) et al., 2007 |
| KNTs | Cd (II) | 116 | Abukhadra et al.,2018 |
| Bluk serpentine | Cd (II) | 16.4067 | Wang et al.,2024  Wang et al.,2024 |
| EX. Ser-nanosheet | Cd (II) | 65.13 |  |
| CTAB/MS  CTAB/MS  CTAB/MS | Cd (II) | 491.9 | This study  This study  This study |
|  | Co (II) | 481.8 |  |
|  | Cu (II) | 434.3 |  |


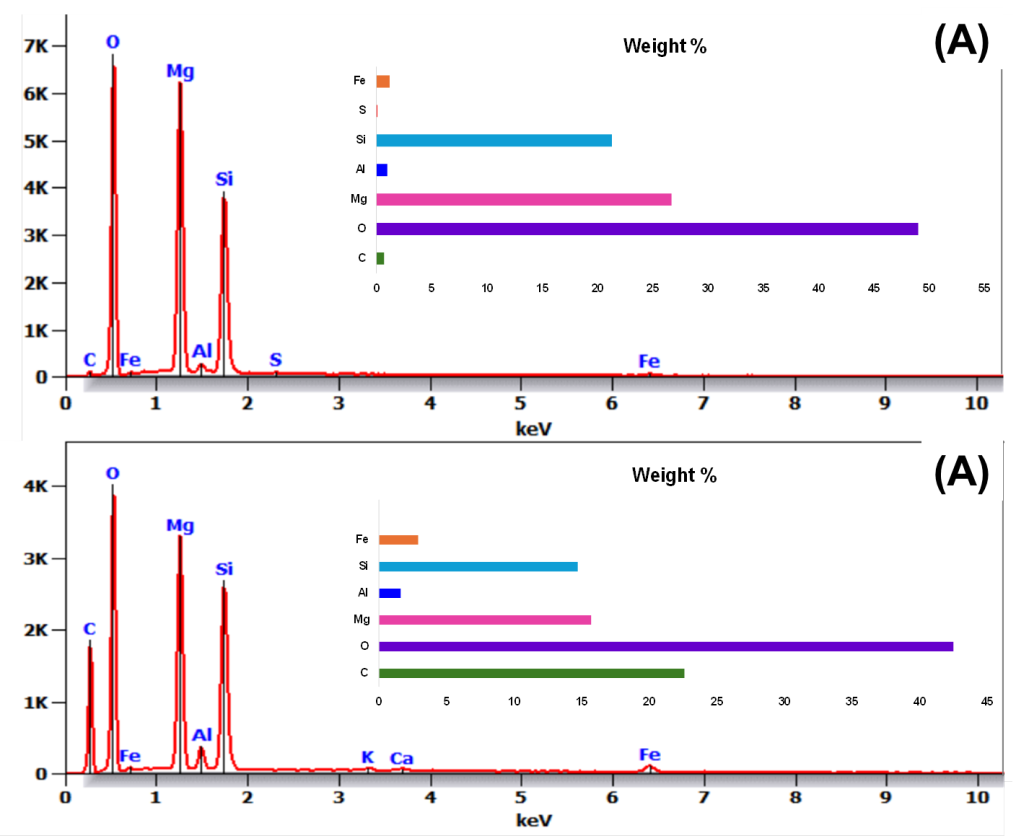


**Fig. S1.** EDX spectra of raw serpentinite (A), and CTAB/MS (B)
